# Supplementary material for: Harnessing population diversity: in search of tools of the trade
Source: Gigascience. 2024 Sep 27;13:giae068. doi: 10.1093/gigascience/giae068 (PMC11427908; doi:10.1093/gigascience/giae068)
Supplement: giae068_GIGA-D-24-00209_Original_Submission [file giae068_giga-d-24-00209_original_submission.pdf]

GigaScience

Harnessing population diversity: In search for tools of the trade

--Manuscript Draft--

**Manuscript Number:**	GIGA-D-24-00209
**Full Title:**	Harnessing population diversity: In search for tools of the trade
**Article Type:**	Review
**Funding Information:**	
**Abstract:**	Big neuroscience datasets are not big small datasets when it comes to quantitative data analysis. Neuroscience has now witnessed the advent of many population cohort studies that deep-profile participants, yielding hundreds of measures, capturing dimensions of each individual's positionin the broader society. Indeed, there is a rebalancing from small, strictly selected, and thus homogenized cohorts towards always larger, more representative, and thus diverse cohorts. This shift in cohort composition is prompting the revision of incumbent modeling practices. Major sources of population stratification increasingly overshadow the subtle effects that neuroscientists are typically studying. In our opinion, as we sample individuals from always wider diversity backgrounds, we will require a new stack of quantitative tools to realizediversity-aware modeling. We here take inventory of candidate analytical frameworks. Betterincorporating driving factors behind population structure will allow refining our understanding of how brain-behavior relationships depend on human subgroups.
**Corresponding Author:**	Danilo Bzdok<br>Montreal Neurological Institute<br>Montreal, QUEBEC CANADA
**Corresponding Author Secondary Information:**	
**Corresponding Author's Institution:**	Montreal Neurological Institute
**Corresponding Author's Secondary Institution:**	
**First Author:**	Danilo Bzdok
**First Author Secondary Information:**	
**Order of Authors:**	Danilo Bzdok
	Guy Wolf
	Jakub Kopal
**Order of Authors Secondary Information:**	
**Additional Information:**	
**Question**	**Response**
Are you submitting this manuscript to a special series or article collection?	No
**Experimental design and statistics**<br><br>Full details of the experimental design and statistical methods used should be given in the Methods section, as detailed in our Minimum Standards Reporting Checklist. Information essential to interpreting the data presented should be made available	Yes

Powered by Editorial Manager® and ProduXion Manager® from Aries Systems Corporation

in the figure legends.<br><br>Have you included all the information requested in your manuscript?	
**Resources**<br><br>A description of all resources used, including antibodies, cell lines, animals and software tools, with enough information to allow them to be uniquely identified, should be included in the Methods section. Authors are strongly encouraged to cite Research Resource Identifiers (RRIDs) for antibodies, model organisms and tools, where possible.<br><br>Have you included the information requested as detailed in our Minimum Standards Reporting Checklist?	No
If not, please give reasons for any omissions below.<br><br>as follow-up to "**Resources**<br><br>A description of all resources used, including antibodies, cell lines, animals and software tools, with enough information to allow them to be uniquely identified, should be included in the Methods section. Authors are strongly encouraged to cite Research Resource Identifiers (RRIDs) for antibodies, model organisms and tools, where possible.<br><br>Have you included the information requested as detailed in our Minimum Standards Reporting Checklist?<br><br>"	review paper
**Availability of data and materials**<br><br>All datasets and code on which the	No

conclusions of the paper rely must be either included in your submission or deposited in publicly available repositories (where available and ethically appropriate), referencing such data using a unique identifier in the references and in the “Availability of Data and Materials” section of your manuscript.<br><br>Have you have met the above requirement as detailed in our Minimum Standards Reporting Checklist?	
If not, please give reasons for any omissions below.<br><br>as follow-up to "**Availability of data and materials**<br><br>All datasets and code on which the conclusions of the paper rely must be either included in your submission or deposited in publicly available repositories (where available and ethically appropriate), referencing such data using a unique identifier in the references and in the “Availability of Data and Materials” section of your manuscript.<br><br>Have you have met the above requirement as detailed in our Minimum Standards Reporting Checklist?<br><br>"	review paper

Manuscript Click here to access/download;Manuscript;Bzdok_v3_240415.pdf

Harnessing population diversity: In search of tools of the trade

Danilo Bzdok[1,2], Guy Wolf[2], Jakub Kopal[1,2]

[1]*MNI – Montreal Neurological Institute, Montreal*
[2]*MILA – Quebec Artificial Intelligence Institute, Montreal*

Abstract
Big neuroscience datasets are not big small datasets when it comes to quantitative data analysis. Neuroscience has now witnessed the advent of many population cohort studies that deep-profile participants, yielding hundreds of measures, capturing dimensions of each individual's position in the broader society. Indeed, there is a rebalancing from small, strictly selected, and thus homogenized cohorts towards always larger, more representative, and thus diverse cohorts. This shift in cohort composition is prompting the revision of incumbent modeling practices. Major sources of population stratification increasingly overshadow the subtle effects that neuroscientists are typically studying. In our opinion, as we sample individuals from always wider diversity backgrounds, we will require a new stack of quantitative tools to realize diversity-aware modeling. We here take inventory of candidate analytical frameworks. Better incorporating driving factors behind population structure will allow refining our understanding of how brain-behavior relationships depend on human subgroups.

Corresponding author: Danilo Bzdok, danilo.bzdok@mcgill.ca

Introduction

To truly understand individual variation in the human mind and neurobiology, future neuroscience studies will need to recognize how modes of population stratification tie into human diversity. 'Mode' here refers to a driving source that is underneath a set of observed variables, without making a statement about the kind of underlying driving forces, which can touch on socioeconomic status[1] or demographic background profile[2]. Each human's brain is shaped by genetic, environmental, lifestyle, socioeconomic, and many other factors, as well as, even more challenging to study, combinations thereof. The human brain is constantly reorganized through sedimentations during years of life experience and nature-nurture interactions. On the other hand, any psychological measure from the brain ultimately depends on individual biological differences that are downstream of individual genetic architecture. In other words, many neural processes are directly or indirectly influenced by one's collection of genetic variants. These factors culminate in vast inter-individual differences. This is why modes of population diversity may often exhibit stronger effects on brain structure and function than the effects of scientific interest that neuroscientists are actually hunting.

To qualitatively appreciate and quantitatively model each participant's diversity background, different areas of neuroscience currently have different attitudes. For example, in the area of common-variant genetics, it is considered standard practice to control for population stratification using principal components derived across allele variants[3–5]. In sharp contrast, this known source of heterogeneity between individuals' background is underappreciated in most neuroscientific studies today. In big and small neuroscience studies, genetic architecture is rarely available or taken into account in the analysis workflow. Yet, the object of investigation, the human brain, is identical in these different research communities. Hence, we wonder whether it can be simultaneously true that genetic studies on topics related to the brain imperatively control for broader differences in genetic architecture, whereas other brain studies, such as those using brain-imaging or behavioral experiments, can safely ignore these same sources of variation that exist between individuals at the population level.

In the age of big data neuroscience[6], we argue here, identifying and embracing diversity factors (e.g., ethnicity, religious belief, diet, height, immigration history, gender identity spectrum, personality, or hormone metabolism) as critical contributors to complex interindividual differences will unlock a step towards diversity-aware modeling in health and disease. This article presents a technical reflection on i) the extent to which today's neuroscience studies are handling population diversity only incompletely, and ii) which kinds of quantitative analysis frameworks could lend themselves to achieving that goal.

Population stratification factors shape what neuroscientists find in their studies

The lion's share of variation in our brain-related measurements (e.g., behavior, brain-imaging, and genetics variants) is not necessarily specific to a neuroscience question under investigation[7]. Strong background variation at the population level is rearing its head at multiple levels when investigating the human brain[8]. In much of human research, generic demographic descriptions like height, weight, and body mass index routinely show stronger effects than the phenotypes that researchers set out to interrogate (Figure 1A). We are only beginning to understand how sociodemographic factors scaffold the mind and brain. We will, therefore, give broader examples in the following of the possible relevance for how to derive insights from future studies in neuroscience.

At the level of genetic variants with direct implications for drug treatment, rs2242480 polymorphism within the CYP3A4 gene is associated with increased warfarin clearance and decreased platelet activation in clopidogrel treatment - one of today's most important drug options for coronary artery disease. This genetic variant is present in 7% of individuals with English and Scottish ancestry but at a frequency of 84% in the Yoruba population in Ibadan, Nigeria, Africa (De et al., 2020). Due to the uneven occurrence of the variant between populations, rs2242480 can only be identified as associated with drug response in cohorts whose allele frequency is high enough to achieve adequate statistical power.

At the level of brain architecture, a line of research demonstrates that humans show a highly reproducible spatial pattern of biological sex differences in regional grey matter volume, including male-biased volumes of the putamen, amygdala, hippocampus, and temporal pole and female-biased volumes of the cingulate, superior parietal, and lateral prefrontal cortices (for review see [9]). As an attempt to account for such differences in brain architecture related to sex and other sociodemographic factors, ABCD recently developed Reproducible Matched Samples that indeed acknowledge the influence of site, age, sex, ethnicity, grade, highest level of parental education, handedness, combined family income, and exposure to anesthesia in studies focused on brain-behavior associations[10,11].

At the level of societal differences measured by socioeconomic status (SES), indicators of material wealth, social prestige, and education are associated with cognitive achievement throughout people's life trajectories[12]. In other words, SES is a central factor at play in cognitive development, educational attainment, and overall health outcomes[13]. Specifically, evolved human abilities, such as spoken language and reading abstract information, are known to differ sharply as a function of SES[14]. By extension, SES shapes individuals' life outcomes by gating access to opportunities and resources, including social capital in the form of social network memberships, nutrition habits, as well as education and exposure to cultural richness[15,16], all continuously remodeling neural systems.

At the level of extraordinary population events, sociological factors such as immigration status, mother tongue, single-parenthood, and experience of racism emerged as the primary contributors to negative experiences during the COVID-19 pandemic. In a data-driven study of >17,000 variables describing ~10,000 families in >20 cities across the USA, social determinants of inequity were found to explain much of the differences in how children and parents experienced the COVID-19 pandemic, above and beyond other candidate predictors such as pre-existing medical or psychiatric conditions[17]. Therefore, dimensions of social inequality played an incisive role in the consequences of the lockdown experience imposing mass social isolation – which should be taken into account in brain-behavior studies with recruiting during COVID-19.

At the level of nutrition habits, the food environment can color and confound study results[18]. As a "natural experiment", several schools in Greenwich, England, participated in a campaign to replace highly processed food high in fat, salt, and sugar with healthier meals prepared from scratch[19]. Providing healthier food resulted in improvements in literacy and science tests. Therefore, nutrition and other environmental factors can introduce skewing into brain-behavior studies, if unacknowledged, thus deteriorating their conclusions' validity and generalizability.

At the geographic level, co-incident geographical variation in genotypes and health traits can bias epidemiological inference from genetic data[20]. Even the relatively genetically homogenous cohort of white British individuals from the UK Biobank exhibit notable north-south differences, including in the

allele frequency of the height-related genetic variants[21] – such geographic trends can color and confound brain-behavior studies.

Given the complexity of interactions between biological, environmental, genetic, lifestyle, and social factors, it is not surprising that major brain disorders (e.g., autism spectrum disorder, schizophrenia, or Alzheimer's disease) exhibit subpopulation-related divergences or even diametrically opposed effects for prevalence, symptoms, and treatment response (Figure 1B). In other words, underlying neurobiological processes and pathways can be tightly interlocked with sex[9], age[22], ethnicity[23], and potentially many social identity factors. While some brain associations (e.g., neuroimaging) might display "small" effects, they might reflect empirically larger differences in finer aspects of neurobiology (e.g., cell counts)[24], which in turn are responsible for the observed differences in disease prevalence and presentation. On its upside, that is why precision medicine has the potential to improve medical care via tailored medical treatments and interventions for individual patients based on their unique genetic and social backgrounds, as well as lifestyle factors[25]. On its flipside, the consequences of a multi-layered population structure pose a pressing challenge to the impactful application of machine learning for precision medicine, such as by standard deconfounding tools, in widespread use today, becoming blunt (Figure 1C). Accurate predictions for individuals across population strata that are fair and do not discriminate against any subgroups will need to be based on methods acknowledging the full complexity of subject diversity.

Deeply phenotyped population cohorts hold the key

There is an urgent need for refined phenotype prediction frameworks due to the increasing realization that sociodemographic factors are deeply intertwined with many or most brain-behavior relationships under common investigation. Further, since disease risk can often emerge from the combination of genetic and environmental phenomena[26], we may not be likely to reveal the mechanisms behind inter-individual and inter-subgroup differences in brain health and disease without a comprehensive coverage of major population-level influences on the brain[8]. Nevertheless, we can only account for nuisance or confounding sources that we have actually measured in our data acquisition and research endeavors. More formally, the ignorability assumption holds that all confounding variables that influence both input variables and phenotype of interest have been measured and are appropriately accounted for in the quantitative analysis[27]. This assumption implies that there is no hidden influence due to unmeasured variables. In other words, all confounders that could affect both explanatory variables and outcomes are included in the analysis model. Since known unknowns, like differences in various demographic dimensions, are not available in many commonly used neuroscience datasets, we risk violating the ignorability assumption (i.e., absence of influence of unknown unknowns on the study at hand). There are now several study cohorts that aim to faithfully represent wider populations. Specifically, the UK Biobank Imaging, ABCD, and other broadly sampled cohorts set out some years ago.

Perhaps for the first time in the history of neuroscience, these collections provide sufficiently detailed and nuanced pictures of each participant's diversity background, including the sociodemographic anchoring in the wider society[28]. Rich phenotypic batteries offer a large number of possible dimensions of diversity that could be taken into account. However, we currently do not have clarity on how to decide what dimensions of population diversity are best to consider when modeling a phenotype of interest. Related but different, neuroscience communities are still at a loss for analytical frameworks identifying

diversity dimensions that drive interindividual variation in small- and big-sample studies alike. That is why population datasets are an untapped asset, or perhaps even a necessary prerequisite, to be in a position to start unraveling the form and implications of major diversity factors for our phenotypes of actual interest[29]. The recent advent of big data in neuroscience[6] thus puts us in a position where we can start developing new methods and frameworks able to identify dimensions of population diversity and incorporate them appropriately in everyday modeling efforts.

Why today's practices for confound variable correction in neuroscience are insufficient

Of note, our present considerations are distinct from and do not try to solve the larger problem of causal inference. Causal notions like 'collider' or 'mediator' can only be applied if a causal graph is available that the investigators are ready to trust (e.g., most mediation analyses are hopelessly reductionist): what set of variables are relevant to consider for a research question at hand and how they influence each other, directionally (more formally, a directed acyclic graph [DAG]) - this is not the state of affairs in many parts of neuroscience and biomedicine (e.g., nutrition science and its relevance for the brain may be especially far away). All here discussed analysis tools can be deployed without such causal graphs.

From a broader epistemological standpoint, very few corners of neuroscience, in particular and biomedicine, in general, possess high certainty about what causal graphs investigators should definitely assume in their research studies. Even more so, few pockets of neuroscience are in a position to draw genuine, clean causal inference, at least as leaders in the technical community define it (Pearl, 2000). Not even GWAS hits pointing to a single protein in the human genome can be routinely declared a causal inference success (e.g., due to a phenomenon called linkage disequilibrium). Not even brain tissue lesion studies in neurological patients can commonly dissect causal effects in primary biology (e.g. because the extent of local grey matter versus passing white matter axon lesion is often unclear). Even broader, to the best of the authors' knowledge, there exists no single causality framework today that can be considered to enjoy support by a majority of scientists - Judea Pearl's[27], Andrew Gelman's[30] and David Blei's[31] views on how causality should be conceptualized are largely diverging; and may perhaps be categorically irreconcilable. For these and many other reasons, neuroscience investigators do not all have unrestricted access to a "click-button" solution to carve out authentic causal links between brain and behavior.

Historically, there has perhaps been an overreliance on convenience-based recruitment of study participants in neuroscience. We inherited our deconfounding practices mainly from a time when our participant samples were homogenous samples drawn from a local geography, a single city, or students from a single university department or patients admitted to only one medical clinic. Applying strict inclusion/exclusion criteria routinely narrows down the observable variability in a target phenotype that, for a fact, does characterize its full form in the general population. Consequently, the overlap in variability between a population under study and the real population was intentionally narrowed down at the behest of the investigator. That is, the more strict the selection criteria, the more the subject cohort is artificially "cleaned" into a potentially under-representative niche of society.

In the example of randomized controlled trials (RCTs), the current medical research enterprise strives to maximize internal validity by excluding participants with comorbidities[32]. Specifically, up to 81% of RCTs published in high-profile medical journals indicate excluded participant subgroups, such as those with multiple chronic conditions[33]. However, neurological and psychiatric patients carrying certain comorbid medical diagnoses may be an authentic aspect that does, in fact, co-occur with a human phenotype in the wider society[34,35]. Therefore, maximizing internal validity might lead to a "keyhole view of the research question", leading up to conclusions of limited traction when facing the whole population.

Importantly, population datasets take a step closer to covering and thus drawing insights from the real, actual human condition. However, a large dataset may not be a big small dataset when it comes to appropriate modeling practices[36]. When acknowledging the full heterogeneity that actually does exist in the population, both old and new nuisance deconfounding strategies appear insufficient at mitigating the wide-ranging consequences of population diversity[37].

Clumsy handling of variation outside of primary scientific interest can lead to spurious associations induced between pairs of otherwise independent variables or biased significance of real brain-behavior associations[38]. In addition, neuroscientists often correct only for linear variation but not nonlinear structure in the data. Hence, more advanced downstream modeling tools, such as many machine learning and deep learning algorithms, may still pick up the complex patterns of nuisance-relevant data variation. Recent evidence documents various nonlinear effects in human population modeling[39]. In the example of predicting a person's age using nuisance-"cleaned" brain measurement, nonlinear age-related bias has been frequently observed: the predicted brain age tends to be estimated as older than the actual chronological age for young participants and younger for older participants[40].

Finally, current deconfounding modeling practices are often idiosyncratic to a particular neuroscience lab or agreed upon in a specific research area. The RNAseq and brain-imaging community typically employ two-step processing[41], first deconfounding and then running a statistical model of interest. On the other hand, in GWAS genetics, confounding variables are part of the statistical model[3]. This approach is not in all cases identical to first removing nuisance variation and then modeling an effect of scientific interest[42]. As we transition to always more representative subject cohorts, in our opinion, neuroscience will need to design new themes of handling variation outside of primary scientific interest and adopt more rigorous practices tailored to investigations of large and heterogeneous samples.

Importantly, several of the proposed candidate tools (cf. below) do not subscribe to the binary black-white decision to "clean" variables from unwanted variation; but, instead, aim at conjoint modeling of variables of scientific interest hand-in-hand with a rich context of known background variation; thus, enabling less categorical statements with greater nuance in brain-behavior modeling. As a classic example, it is a widespread practice in neuroscience and biomedicine to remove or account for age- and sex-related variation in quantitative analyses. Instead, what we are arguing here is that there may be value in the more multifaceted treatment of probing brain-behavior relationships *as a function of* age/sex-related variation. This modeling agenda turns sources of background variation into additional variables of interest. Appropriate candidate tools would be able to thrive on the full breadth of participants' diversity backgrounds; going well beyond the covariates of age, sex and perhaps socioeconomic status that are commonly available in many biological or medical datasets. Taken to its extreme, it may turn out that *different* brain-behavior relationships require investigators to consider a *different* set of most relevant sources of population variation. In short, there may be no such thing as a definitively "cleaned variable" for any circumstance or any research question.

Lessons from genetics

Legacy deconfounding approaches and practices in neuroscience have maybe been passed on from one generation of investigators to the next generation, maybe sometimes uncritically, in an era of boutique in-lab experiments and before the age of big-data neuroscience. These simple techniques are in danger

of turning blunt when hunting for effects smaller than the effects driven by major sources of population stratification.

When considering neighboring fields that underwent expansive growth in subject sample sizes, genetics can serve as an example. The sample sizes of genome-wide association studies (GWAS) increased almost 300-fold over the last 15 years – from the first large, well-designed GWAS for complex diseases with a total of 17,000 participants[43] to more than 5 million people in the current largest existing GWAS aimed at predicting height[44]. As an immediate consequence relevant to our present considerations, the analysis of millions of individuals will be increasingly based on an admixed population with many ties between layers of population structure, including genetic background. The heterogeneity of inherited genetic backgrounds in association studies may lead to false-positive or false-negative findings[45].

The chopstick gene study serves as a thought experiment[46]: A hypothetical study sets out to explore the ability to eat with chopsticks in the admixed population of people with Chinese and European descent. GWAS targeting the ability to use chopsticks using individuals across major population groups would identify several candidate genes, including the human leukocyte antigen (HLA) system. The allele HLA would be associated with the ability to use chopsticks not because immunological determinants play any role in motor dexterity but simply because the allele HLA is more generally common among people with Chinese and European background; independent of the study goal. Traits present at a higher frequency in an ethnic group are likely to show a notable association with alleles that also happen to be more common in that group[45]. Such collinearity effects will need to be considered in future neuroscience studies to guard against spurious brain-behavior findings.

To adjust for false positive hits due to alleles occurring at different frequencies across populations, prior genetic investigations often narrowed the scope of ancestries under study. In addition, the GWAS community was looking for an analytical solution able to account for the effect of ancestry, which would be convenient, interpretable, and, importantly, scalable to millions of individuals. Among several candidate approaches, the community took the collective decision to control for population structure by adjusting for a set of covariates derived with singular-value-decomposition-based methods[4,5]. Principal Components Analysis is a tool that has been used to infer population structure in genetic data for several decades[47]. According to current GWAS practices, the top principal components (PCs) are typically used to adjust for population stratification. These PCs, derived across thousands of genetic variants, may not always only reflect population structure. Specifically, some PCs might also partially track family relatedness, long-range linkage disequilibrium (for example, due to inversion polymorphisms), and differences in laboratory treatment or assay artifacts[48]. However, even if the overall genetic composition (global fraction) is identical between cohorts, individuals' local ancestry makeup may differ (e.g., in populations with multiple ancestral groups, such as individuals with mixed European, African, and Indigenous American ancestry in the Americas). PCs capture a larger variety of admixtures but may not capture this local ancestry makeup of individuals[49]. Therefore, current deconfounding approaches based solely on adjusting for top PCs still leave open the possibility for false positive associations and hurting statistical power to detect truthful effects.

What once was a textbook example of genetic evidence for population adaptation through small changes in allele frequencies at hundreds or thousands of loci turned out to be an example of bias due to uncorrected population stratification[50]. Height is a complex polygenic trait estimated to be modulated by as much as 4% of the hundreds of thousands of studied genetic loci[51,52]. Height also has influence on brain-

related measurements, such as brain-imaging recordings using MRI. A series of articles reported evidence of frequency changes at alleles associated with height differences in Europeans driven by specific selection pressures[53,54]. However, signals of genetic adaptation based on large numbers of genetic loci below genome-wide significance are susceptible to biases due to uncorrected population structure. Much of the weak P values are dominated by the effect of residual population structure – any remaining population structure or ancestry-related differences in a study population that has not been adequately accounted for or corrected[21]. Therefore, polygenic adaptation on human height now makes a particular case example where uncorrected population structure in GWAS can influence studies of complex traits via intermingling with major sources of population structure. This example adds to the catalog of evidence that "button-click" corrections of population structure in GWAS may not always be judicious[50] – we can probably do better, but we need to figure out how to do that exactly.

Comparing geographically varying phenotypes, such as height, across cohorts with different diversity backgrounds and environmental conditions can be challenging due to the sources of confounding that are difficult to model. These confounding factors form a web of mutually related genetic and environmental divergences, differences in environmental exposure, gene-gene interactions, gene-environment interactions, historical population size dynamics, and other unknown factors ("unknown unknowns"). Currently, we have not completed our quest for native modeling frameworks to incorporate complex population structures and environmental heterogeneity. Biases at individual loci may appear tiny, but they compound if aggregated across thousands of loci – as manifested in polygenic risk scores pooling across the genome[55]. Therefore, the magnitude of effects due to uncorrected residual stratification is currently hard to quantify[56]. The most direct way to avoid biases associated with population stratification is through genetically homogeneous populations. As such, ancestrally mixed and populations of non-European descent are frequently excluded from associated genetic analyses[49]. This lack of diversity in existing, well-powered brain-imaging or genetic datasets probably arose as a result of both scientific and logistical challenges, such as difficulty recruiting participants from minoritized populations. In the US, admixed groups make up more than ten percent of the population, with the ratio continuously increasing over time[57]. The development of scalable methods that allow correct statistical genomic prediction for admixed groups independent of genetic background will boost the clinical utility of large-scale data-collection efforts for minorities and help reduce existing health disparities.

Candidate tool: Propensity score modeling

Failing to acknowledge relevant sources of variation can give rise to bias and unfairness. In the example of disease-control comparison, manually aligning participants based on age and sex is probably one of the most common matching tactics in neuroscience today. However, this manual approach to balancing out subject groups becomes increasingly impractical with every additional covariate of undesired variation. Such person characteristics may have different forms: binary yes-no (e.g., drug usage), ordinal (e.g., neighborhood deprivation brackets), and continuous (e.g., exposure to green spaces). Propensity scores (PS; Figure 2, Suppl. Box 1) have the appeal of summarizing a potentially large number of heterogeneous person characteristics into a single balancing score (with respect to a target phenotype of interest). Initially conceived by Rosenbaum and Rubin[58] for comparing target treatment and control groups, PS is defined as the probability of treatment assignment conditional on several available person characteristics:

$$PS = P(y = 1|C),$$

where y is the outcome or target phenotype of actual scientific interest (e.g., treatment versus control), while C denotes a collection of covariates (e.g., an array of subject diversity indicators) to be accounted for. All subjects with and without phenotype y that map into the same PS range are made comparable or balanced out with regards to the covariate set C, with some analogy to how randomized clinical trial experiments are conducted. If one of the covariates does not actually explain the target phenotype, we do not need to balance the group subjects according to that source of population variation.

Capitalizing on the obtained PS, the distribution of measured characteristics can be homogenized to maximize the overlap between the subjects from each group. Scores are computed based on those subject characteristics (C) that we aim to equalize in our to-be-matched individuals. Computationally, PS take the common form of predicted probability derived from the PS model. Several kinds of quantitative models have been examined for PS estimation, including bagging or boosting[59,60], recursive partitioning or tree-based methods[59,61], and neural networks[61]. However, logistic regression is probably the most used implementation. This choice of method yields the following model specification for the PS framework:

$$logit(y) = \beta_0 + \beta_1 C_1 + \beta_2 C_2 + I + \beta_k C_k,$$

where k denotes the number of simultaneously considered sources of population variation C, the intercept β_0 captures the average classification log odds, while $logit(\cdot)$ is a nonlinear transformation (called "link function" in the generalized linear modeling family) that maps from probability space to log odds space.

Importantly, PS estimated using a conventional logistic regression model are based solely on the participant characteristics, that is, without access to input variables of primary scientific interest (such as brain region volumes or gene information coming into play in the subsequent downstream analyses). This minimal reliance on subsequent data analysis steps enables broad applications for PS, such as for the purposes of matching, stratification and inverse probability weighting as well as regression adjustment[62]:

1. PS matching entails forming matched sets of subjects that share a similar value of the PS[58,63]. Among many options, the most common implementation of PS matching is one-to-one matching, in which pairs of subjects from each group are formed such that aligned subjects have similar PS values.
2. Stratification by means of PS involves clustering subjects into mutually exclusive population strata based on their estimated PS. A common approach is to divide subjects into several equally sized clusters, such as by using the quintiles of the estimated PS. These subject strata or "chunks" then represent sets of participants with homogeneous background profiles with respect to the observed covariate set. Hence, building strata of most similar population architecture may require leaving out certain subjects from further analysis due to insufficient overlap in person characteristics.
3. Inverse probability of treatment weighting (IPTW) uses PS to balance measured subject characteristics distribution between groups of interest. In other words, IPTW creates an "artificial" population in which measured characteristics exhibit congruent distribution across groups[64–67]. Multiplying individuals' measurements by their corresponding weight effectively gives more importance to under-represented individuals and less importance to over-represented individuals.

4. During regression adjustment, PS can be included as a nuisance variable in a regression model quantifying the scientific effects of interest. Doing so adjusts the regression coefficients for undesired population heterogeneity.

Pros. As a key asset, the PS framework purposefully precedes the actual core analysis, such as predicting outcome information from brain scans or gene status[68]. Put differently, the weighted contribution of each diversity factor in the PS is estimated solely based on their ability to predict the target phenotype under study from person characteristics. Furthermore, PS can similarly handle both continuous (e.g., age) and categorical (e.g., site or sex) kinds of confounding covariates in a single coherent framework to provide a one-number covariate summary. Any possible mutual correlations (collinearity) in the coefficient estimation of the PS model do not affect the ensuing scores because this modeling step follows the prediction goal[6,69]. Finally, the possibility for using more sophisticated PS models such as random forest and deep learning algorithms for refined PS estimation allows for incorporating high nonlinearity (square or cubic terms of characteristics and convoluted interactions between characteristics) among the sources of population diversity.

Cons. As an ensuing disadvantage, PS can only balance the observed subject characteristics available to the investigator. Some imbalances may remain even after PS adjustment if relevant subject features were not measured, were measured imprecisely, or were not measurable (cf. ignorability assumption). Moreover, matching on observed variables may infiltrate bias due to dormant unobserved confounders[70]. In addition, PS approaches, especially their nonlinear variants, can be data-hungry, requiring larger sample sizes to be estimated correctly.

Candidate tool: Bayesian hierarchical modeling

Additionally, data on human brain and biology commonly exhibit rich, native multilevel structure. This nested structure is inherent to measurements of individuals, brain recordings, or genes that can cluster based on sex, lifestyle markers, work-life markers, as well as time, place, and context of measurement. Such a highly structured dataset invites an explicit hierarchical description in our quantitative modeling efforts that appreciates data organized in a cluster-segregated manner. Bayesian hierarchical modeling (BHM; Figure 3, Suppl. Box 2) represents one of the best statistical frameworks to seamlessly integrate multiple levels of information in a coherent analysis approach. BHM, also called multilevel modeling, combines Bayesian analysis with a pre-specified hierarchical structure to carefully tease apart variation within and between sources of population diversity. Hierarchical estimation of parameter values allows for the adaptive, interlocked modeling of group-level and individual-level variability with regard to diversity phenotypes realized via regression parameters that can vary by sources of population variability[71].

The Bayesian flavor of the multilevel structure comes into play via distributions for each model parameter and for parameters governing those parameters - hyperparameters[72]. Specifically, pre-specified domain knowledge (such as about population strata), as well as uncertainty around it, is articulated as prior distributions assigned to the parameters at each level of the hierarchy (before the model “sees” any data). During model parameter value estimation, the measured participant data at different levels are then used to update these prior distributions to obtain the ensuing posterior parameter distributions, reflecting the obtained revised knowledge. Using hyperparameters and nested

structure encourages model parameters across different groups to "talk to each other", thereby forming better overall parameter estimates for all groups or population strata[73].

By pooling and propagating information across sources of variability, the data from all individuals feed into the higher-level parameter distributions (hyperparameters), which in turn percolates into the estimates of each individual lower-level parameter. This natural consequence of hierarchical structure (cf. shrinkage) results in lower (data-) level parameters guided by the overarching population-level parameters during model estimation[74]. In light of observed data, estimates for copiously sampled groups will share statistical strength with under-sampled groups, thus drawing estimates toward the group-wide average. On the other hand, parameter value estimates for groups with larger subject sample sizes might depart more from the group-wide average[75]. The appropriate amount of this shrinkage is learned adaptively from the data themselves[76].

As an illustrative example of multilevel structure, BHM in the form of linear regression with varying slopes and varying intercepts quantifies the relationship between an input variable (x) and an output variable (y) while accounting for group-specific variation (population strata such as cities or countries):

$$y_{ij} = \beta_{0j} + \beta_{1j}x_{ij} + \varepsilon_{ij},$$

where β_{0j} and β_{1j} are group-specific intercept and slope parameters, respectively, while ε_{ij} captures the individual-level error term pertaining to observation i and group j.

The group-specific (or data-level) parameters are, in turn, modeled using governing hyperparameters:

$$\beta_{0j} \sim Normal(\mu_{\beta_0}, \sigma^2_{\beta_0})$$
$$\beta_{1j} \sim Normal(\mu_{\beta_1}, \sigma^2_{\beta_1}),$$

where μ_{β_0}, μ_{β_1} are the higher-level mean values for the group-level intercept and those for the slope parameters, while $\sigma^2_{\beta_0}$, $\sigma^2_{\beta_1}$ are the variances of the group-level intercept and slope parameters, representing the degree of dispersion among the group-dependent model parameters in the higher model echelons (above the bottom data level). All these hyperparameters come with specified prior distributions.

The presented model simultaneously estimates the overall relationship between y and x as well as the variations in this relationship between different groups. The higher-level parameters (hyperparameters $\mu_{\beta_0}, \mu_{\beta_1}, \sigma^2_{\beta_0}, \sigma^2_{\beta_1}$) estimate a baseline for a cohort (e.g., across-city effect), whereas the group-specific deviations from that baseline (e.g., city-by-city effects) are estimated by the lower-level model parameters. Hence, BHM components are fit differently to different segments in the population under study.

There are four common and complementary reasons to use multilevel models for population modeling[76]:

1. Adjusting estimates for imbalance in subject sampling. When some individuals, locations, or times are sampled more than others, shrinkage protects parameters from being affected by random sampling noise. Shrinkage in hierarchical models pushes low-level parameters to shift their estimates toward the higher-level parameters' distributions. Therefore, even one observation in many groups is sufficient to fit a multilevel model[72].
2. Adjusting estimates for repeated sampling. When more than one observation arises from the same individual, location, or time, failing to explicitly account for the correlations between

repeated samples leads to biased estimates. BHM effortlessly models repeated measurements for the same individuals in different contexts or time points via a hierarchical structure, where individual-level factors explain changes over time, even if measurement repetitions are incomplete or otherwise heterogeneous.

3. Breaking down interindividual variation as a function of group characteristics. BHM is an ideal framework to batch coefficients as a function of sources of variation to directly specify and model possible stratification in the cohort, thus affording the ability to estimate meaningful quantities from a diversity standpoint. Several groups and group characteristics can be modeled simultaneously and against each other.
4. Avoiding averaging (such as for each population stratum). Any averaging of values discards some potentially rich sources of interindividual variation. BHM goes beyond simple averaging by considering the variability and structure of the data at different levels, resulting in more accurate and nuanced insights. Hence, BHM allows for preserving the uncertainty in the measurements while still using the average to make predictions.

Pros. Classical linear regression deconfounding models ascribe no structure to the parameters and try to remove (orthogonalize) the parameters of scientific interest from unwanted nuisance sources. In contrast, BHMs are “massive interaction engines” that draw on the hierarchical data structure to jointly model various heterogeneous sources of variation together with the effects of interest. Hence, BHMs can state and appropriately handle sources of variability interactions nested within multiple levels. In addition, principled quantification of uncertainty through the Bayesian framework offers clean contours of confidence rarely available in non-Bayesian approaches. Finally, via shrinkage, BHM negotiates the tradeoff between underfitting abundantly sampled and overfitting under-represented population strata[72].

Cons. As is true for all statistical models, the parameter estimates are meaningful descriptions of the data only in the context of the model structure pre-specified by the investigator. Due to the flexibility and module-like structure, each BHM specification needs to be hand-tailored to each particular setting and research question, which requires technical and mathematical expertise in the project team. Therefore, along with the mathematical and computational demands, BHMs build on a strong conceptual understanding of hierarchical nested relationships (i.e., an assumed data story of how the observations came about)[77]. In other words, BHMs pose both the opportunity and the challenge of integrating additional assumptions, compared to classical regression, since each modeling level is its own (component) model with assumptions[78]. Due to this complexity, interpretation can be more demanding than in traditional regression models. No single number, metric, or summary will authentically capture the behavior of a BHM system.

Candidate tool: Multilevel regression and post-stratification

Insufficiently representative study cohorts are a common barrier to materialized progress in the neurosciences. Historically, scientific knowledge about the human brain was often based on findings from small homogenous, sometimes over-selected samples of Western, educated, industrialized, rich, and democratic societies[79]. These samples with sample-population discrepancies were the results of convenience sampling, where participants are recruited for a study based on their ease of accessibility and availability. Despite sampling biases, obtained findings were often assumed to reflect universal

aspects of brain structure and function. To confront such imperfection of many cohort datasets in political polling and election outcome prediction, Gelman and Little[80] introduced a multilevel regression with a post-stratification (MRP; Figure 4, Suppl. Box 3) framework able to adjust findings for discrepancies between the study sample and the general population. As an opportunity for horizontal knowledge translation, MRP offers overlooked solutions to neuroscientists by improving the representativeness of predictions in under-represented and under-sampled population segments[81].

The key idea behind this statistical technique is to use external knowledge, such as census surveys in the USA or Canada, about population strata to help de-bias the estimates of the multilevel regression model[82]. In the first step, a hierarchical multilevel regression model is built to estimate the outcome of interest given the input variables and a set of person characteristics (e.g., sex, ethnicity, age, education, but also religious belief, ideology or other life factors). In the next step, the cohort participants are partitioned into a grid of disjoint strata (cells) based on combinations of the various person characteristics. Since the person's characteristics might contain continuous variables (e.g., age, income), these variables need to be split into intervals to create a finite number of categorical variables. Afterward, the multilevel regression model estimates the outcome of interest within each cell. The benefit of the hierarchical structure is that knowledge from well-sampled groups is pooled to improve parameter estimation for the under-sampled groups (cf. Bayesian hierarchical models). Consequently, estimates for relatively sparse cells are improved by "borrowing strength" from demographically similar cells with richer data[83].

In the second step, each cell-level estimate is weighted by the relative known proportion of subjects with that particular set of person characteristics learned from external knowledge about the full population. This process to account for the discrepancy in sampling is known as "poststratification". In particular, the poststratification for the population outcome of interest $\hat{y}$ is calculated as the weighted average of cell-wise posterior estimates where the weighting scheme is determined by the size of each cell in the population:

$$y^{MRP} = \frac{\sum_{j=1}^{J} N_j \hat{y}_j}{\sum_{j=1}^{J} N_j},$$

where $\hat{y}_j$ is the estimate in cell j and N_j is the size of the jth poststratification cell in the population (external knowledge).

Similarly, outcome estimates at any slice of the population s can be derived as:

$$y_s^{MRP} = \frac{\sum_{j \in J_s} N_j \hat{y}_j}{\sum_{j \in J_s} N_j},$$

where J_s is the subset of all poststratification cells that comprise s.

These new estimates provide a more accurate reflection of the entire population, accounting for the known population characteristics. In other words, poststratification rebalances and "un-skew" the stratum-specific predictions by correcting for the particular sampling demographics.

Pros. MRP unites the strengths of both multilevel regression and poststratification. Multilevel regression efficiently accounts for the hierarchical nature of a cohort dataset. At the same time, poststratification adjusts for population characteristics. Consequently, even highly non-representative samples can yield accurate estimates of population-level attributes. Compared to other non-hierarchical poststratification models, MRP allows using many more categories and, thus, better approaches to representative estimation of population characteristics. The practical gains from this method are greatest

for small subgroups of the population. Finally, MRP circumvents challenges related to study consistency when comparing studies based on cohorts with different diversity backgrounds.

Cons. Since MRP shares the strengths of hierarchical multilevel models, it also shares their weaknesses. MRP assumes that the chosen multilevel regression model accurately represents the underlying relationships in the observed data. In addition, in order for the poststratification to be effective, MRP relies on the availability and accuracy of external information (population characteristics). Therefore, in principle, MRP is limited to using individual-level variables present in both the study and the external source (i.e., census). In addition, if certain population groups are underrepresented due to non-response (but see Figure 2 for recruitment bias correction), the poststratification adjustments may not fully correct for these issues. Finally, MRP introduces an additional layer of complexity to multilevel regression modeling, which itself requires a deep grasp of the specific research context and mathematical modeling.

Candidate tool: Machine-learning algorithms for representation learning in complex data

Computational paradigms for analyzing biomedical data, shaped by population diversity factors, have been greatly influenced in recent years by the progress made in geometric and topological representation learning (Figure 5, Suppl. Box 4). A key observation in this field is that collected data features often contain redundancies and dependencies, but these can be nonlinear and difficult to evaluate or leverage directly. On the other hand, modern machine learning methods (especially deep learning ones, but not only) often implicitly learn latent variables and base their results (e.g., in predictive and generative tasks) on them rather than direct computation from raw input features. In the context of data exploration to uncover driving sources of population stratification, recent developments have provided a toolkit of methods that seek to capture a low-dimensional intrinsic data geometry to uncover emergent structures, patterns, and trends in data while expressing them in interpretable ways. Indeed, while data may seem high-dimensional in the ambient observable space (i.e., the entirety of measured variables), the viable configurations of features from which the data is sampled actually form a low-dimensional non-Euclidean structure that is mapped to the observable space. Further, in biological processes, which evolve gradually over time, with limited degrees of freedom in each short-time step, collected data can be effectively modeled as sampled from locally low-dimensional neighborhoods that smoothly vary to form a nonlinear organization in the high-dimensional feature space. Such inherent structure in data is typically called a *data manifold*, and the assertion that data can be modeled by it is referred to as the *manifold assumption*, giving rise to the field of *manifold learning*. In other words, manifold learning can facilitate a deeper understanding of population variation by uncovering hidden diversity patterns.

In order to leverage the manifold assumption of rich, high-dimensional data (e.g., brain-imaging, common-variant genetics, single-cell genomics), a common starting point is to define a notion of locality in data, encoded by similarity or affinity between data points. Popular visualization methods, such as tSNE and UMAP, directly leverage this notion to preserve the extracted neighborhood structure in a 2D representation that can be easily visualized via a scatter plot. While derived in different ways – tSNE aims to preserve the degree to which points are neighbors in the high- and low-dimensional representations. Instead, UMAP aims to preserve symplectic topology – they both result in equivalent embedding approaches, which can be expressed, such as in terms of attractive and repulsive forces. However, while neighborhood preservation is effective for preserving certain patterns (such as clustering) in data, this

attitude towards data analysis does not directly consider the global data geometry or long-range nonlinear relations in the measured variables. To take these into account, diffusion geometry methods, such as PHATE[84] and its variants, consider connectivity defined by a Markovian random walk (i.e., diffusion process) over a graph defined by the data neighborhoods. In particular, PHATE uses diffusion distributions from each data point to construct potential distances between them and multidimensional scaling to optimize low-dimensional coordinates (e.g., 2D or 3D for visualization purposes), forming Euclidean distances that match those. The construction of potential distances can also be extended to consider a wider family of diffusion information distances, which also encompass certain divergences as well as the traditional diffusion distance introduced in diffusion maps together with the concept of diffusion geometry. By compressing and parsimoniously re-expressing data onto a lower-dimensional space, diffusion geometry can reveal how different individuals or samples are related and how they contribute to the overall diversity in the dataset, aiding in the identification of subpopulations of data points or data clusters with distinct characteristics.

In addition to enabling representation learning, the diffusion geometry construction also enables a native framework to process signals over the intrinsic data geometry it captures. In particular, such methods can be used to provide a low-pass smoothing filter, which can then be applied to data features to denoise and remove collection artifacts from them. For example, this is the key component in the well-known MAGIC[85] data imputation method. It was also used in SUGAR[86] to fill in sparse regions of the data manifold with synthetically generated cell profiles along its geometry. Further, the application of such filters is not limited to measured features and can also be applied to external signals, such as labels of control groups compared to perturbed conditions, as done in MELD[87] in order to estimate the likelihood of cellular profiles originating from specific perturbed populations. Finally, iterative applications of smoothing filters provide gradual coarse-graining of the data, with a time-inhomogeneous diffusion called a condensation process[88]. This process is used in multiscale PHATE[89] to provide an extensive visualization that scans a full range of resolutions to explore and analyze biomedical data. In combination with the related tools mentioned above, multiscale PHATE thus provides an efficient insight into the complexity and heterogeneity of diverse populations.

In summary, recent algorithmic innovations can be used for the purpose of explicitly modeling population diversity. However, as of now, these algorithms may turn out to be more useful for charting major sources of population stratification by visualizing it (e.g., using tools such as PHATE) rather than directly accounting for confounding influences in analyses of primary scientific interest. Put differently, machine learning algorithms may turn out to aid, especially in discovering the form and extent of the still under-explored major sources of population stratification by useful compressions of complex nonlinear relationships.

Direct comparison of candidate tools

Crucially, only model class 4 (high-dimensional visualization) is aimed at discovering major sources of population stratification in the first place. Instead, model classes 1-3 are helpful if the investigator is already aware of what key dimensions of population variation should be examined as relevant background variation: propensity scores (model class 1) aiming to essentially partition the whole participant sample into covariate-set-equivalent subgroups to carry out a subsequent covariate-unaffected actual analysis of interest (two-step procedure), 2) Bayesian hierarchical modeling (model class 2) aims to explicitly model

brain-behavior with their conjoint covariate effects, including detailed uncertainty descriptions therefore, without discarding any information as “nuisance” (one-step procedure), while 3) post-stratification based multilevel modeling (model class 3) aims to alleviate systematic skewing of brain-behavior predictions due to known under- or over-sampling of participant subgroups (two-step procedure).

In terms of technical implementation of these approaches, the high-dimensional structure discovery goal (model class 4) can be achieved with a large variety of dimensionality-reducing tools, including linear (e.g., PCA, including its probabilistic and Bayesian variants) and many more recently emerged non-linear (e.g., t-SNE, UMAP, PHATE, autoencoder neural networks) methods. Propensity scores (model class 1) are commonly realized by logistic regression models but can be extended to any approach deriving an outcome prediction from input variables, in principle. In contrast to frequentism (particular “pre-canned” tools for particular pre-existing use cases), Bayesian hierarchical modeling is a framework to express and build all sorts of model formulations, like putting together lego bricks, to closely adapt to all sorts of use cases, based on a unified, coherent conceptual foundation.

Conclusions

While data resources are reaching the point of being representative of the broader population, our modeling techniques are blunt tools when it comes to handling this increasing complexity. There may be a kaleidoscopic multitude to a given brain-behavior relationship at hand, as we start to view these through the prism of population diversity. This nuanced knowledge will be an unavoidable milestone on our path towards single-subject statements that are fair and unbiased.

The very advancements intended to appreciate sources of diversity in neuroscientific data end up creating new methodological challenges that require additional solutions. As we pull subjects from always more geographies, sociological pockets, and diversity backgrounds, the inconsistencies in what sources of major population stratification are acknowledged and integrated explicitly into our everyday modeling practices become center stage. If heterogeneity in genetic makeup wields a notable influence that geneticists feel prompted to account for, how come many neuroscientists safely ignore it? After all, given that the brain structure and function bear downstream effects from the genetic makeup of a human individual? This consideration demonstrates that we need to audit and edit legacy deconfounding practices that may turn out to be relicts from the previous era of analyzing small homogenous boutique datasets. Only when we are able to endorse and explicitly model diversity backgrounds, we will be able to uncover the entire mosaic of human neurobiology.

Identifying and adopting analysis tools incorporating driving factors of population structure will allow for a refined understanding of how brain-behavior relationships depend on human subgroups. We have made several guesses how diversity-aware modeling could be implemented in future neuroscience studies: Propensity scores enable single-number summaries of heterogeneous person identifiers, which allows matching participants for heterogenous sources of population stratification. Bayesian hierarchical modeling could be useful to neuroscientists who wish to explicitly quantify the extent to which brain-behavior relationship are interlocked with several layers of sociodemographic subgroup variation. Future neuroscience studies may also benefit from implementing post-stratification frameworks that can address an imperfect sampling of subgroups in study cohorts to de-bias estimates of brain-behavior relationships. A key contribution from the machine-learning community may consist in advanced visualization to better understand complex population patterns in a way that is more accessible to neuroscientists.

Figure 1. Unattended sources of population diversity overshadow subtle brain-behavior links studied by neuroscientists

A) Population structure within Europe. Principal components analysis on genotype data from 197,146 loci in 1,387 European individuals produces a low-dimensional summary of the observed genetic variation. Visual representation of the first (PC1) against the second (PC2) principal component bears a notable resemblance to a geographic map of Europe. Small colored labels represent individuals, and large colored points represent each country's median PC1 and PC2 values. The inset map provides the key to the labels. The PC axes are rotated to emphasize the similarity to the geographic map of Europe. This visual summary emphasizes that when mapping the genetic basis of a disease phenotype, spurious associations can arise if the genetic structure is not adequately accounted for. Adapted from Ref[90]. B) Potential biases in clinical predictive modeling. A PubMed search for original neuroimaging research published between 1983 and January 2016 highlighted 475 human clinical studies that included 615 multivariate pattern recognition frameworks. Most studies have focused on diagnosis, identifying brain signatures that discriminate patients from healthy controls. Diagnostic classification accuracy is plotted as a function of sample size for six types of disorders. Across disorders, very high classification accuracy is reported in some small studies, while accuracy values for large-sample studies are much more modest. The high accuracies in smaller clinical studies raise a warning sign of potential optimistic bias due to data dependence and overfitting. Adapted from Ref[91]. C) Established and new deconfounding strategies insufficient to counteract escalating population diversity. Regularized logistic regression with the l2-norm classified between autism spectrum disorder versus typically developing participants with no history of mental disorders based on brain imaging features (e.g., functional connectivity). Stratifying the cohort using propensity scores delineates the impact of diversity on classification performance. Diversity denotes the mean absolute difference in propensity scores between the participants in the training set and those in the held-out stratum. Performance is measured by F1 score on the original brain imaging data (Raw, top) and after carrying out deconfounding steps on the brain feature prior to the pattern classification pipeline using standard linear regression–based deconfounding (LinReg, middle) and recently proposed hierarchical regression framework to control for site differences (ComBat, bottom). The first column shows the model prediction performance using a 10-fold cross-validation strategy. The second column, for each cohort, displays the performance in the holdout participants. Both ComBat and linear regression–based deconfounding failed to mitigate the impact of diversity on prediction accuracy. Adapted from Ref[37].

Figure 2. Propensity score modeling can be used to mitigate healthy volunteer bias

This practical example is based on the UK Biobank (UKBB) cohort and a representative sample from the Health Survey England cohort (HSE) that include 14 common variables (y-axis), tapping into health, lifestyle, education, and basic demographic dimensions, being available in both population datasets. Left) Charting the extent of healthy volunteer bias in UKBB: 14 univariate logistic regression models (single-variable analysis, this is the PS model) were separately used to predict UKBB participation as model outcome (encoding: HSE = 0, UKBB = 1) for each of the 14 characteristics (input variables) within a cohort of 393,299 UKBB participants and 21,816 participants from the representative HSE sample. Right) Weighting subjects accommodate healthy volunteer bias: LASSO regression (multi-variable analysis) was designed to predict UKBB participation (HSE = 0, UKBB = 1) using 903 predictors (14 variables and all possible two-way interactions). The obtained probability weights for each UKBB participant were calculated as the inverse predicted probability from the LASSO model. The application of the probability weights to UKBB participants enabled the creation of a (weighted) pseudo-sample of the UKBB that is more representative of its (representative) target population (HSE). The generated subject-wise weights were applied in the previously used 14 univariate logistic regression models (y-axis). UKBB individuals were given their normalized weight, and HSE participants were given a weight of 1. Previously significant standardized regression coefficients became non-significant, suggesting successful mitigation of healthy volunteer bias (x-axis), as a concrete demonstration of what PS tools can achieve. Adapted from Ref[92].

Figure 3. Bayesian hierarchical modeling can delineate the variability in predicting motor recovery in stroke patients across six separate studies

This practical example shows, in 385 acute stroke patients, how upper limb impairment at the initial (acute) stage can forecast follow-up performance (3-6 months after the brain insult) as indexed by the Fugl-Meyer (FM) scale as a clinical measure of motor recovery after stroke. The goal is to pool knowledge across patient-level information from six different studies on motor rehabilitation while explicitly modeling sources of variability such as slight difference in treatment regimes. The use of Bayesian hierarchical linear regression with varying intercepts and slopes allowed a conjoint analysis pooling information from stroke outcome data from the six different studies. After drawing Bayesian inference on posterior parameter distributions, the relative contribution of each of the six studies was characterized by fully specified probability distributions (credibility intervals) gauging the effects of the intercept parameter (alpha, effectively estimating the average recovery outcome) and slope parameter (beta, estimating how initial predicts later motor impairment). This approach answers the question how much information propagated across studies agrees or disagrees with each other, in a continuous fashion, by virtue of the inferred posterior hyperparameter probability distributions; rather than blindly merging participant data from all six studies, acting like their participant information come from a same source. A) Full model specification: dependencies between variables are indicated with arrows, observed variables are in grey boxes, and the prior distribution defining variables are shown on the right. M denotes the number of studies analyzed and $N(i)$ the number of subjects in each study. B) Aggregated motor study posterior effects in two different notations: the relationship between the across-study intercept and slope hyperparameters is depicted in a two-dimensional density plot. The across-study intercept captures the average motor effect, and the across-study slope captures performance gain dependent on the initial motor impairment as adaptive overall effects across study sources of variability. C) Individual motor study posterior effects: The joint density for plausible combinations of intercepts and slopes highlights the relationship between sample size and credibility interval width, given the visited data of the 6 included studies. We see that higher-sample studies led to narrower confidence contours (see legend in C for study-specific color coding). This use-case illustrates how Bayesian hierarchical modeling tools can be used for the purposeful quantification of the within- and between-study variability in recovery outcome prediction in upper limb motor impairment several months after stroke – the core point being that what is usually regressed out as a site difference covariate is here directly integrated into the model of scientific interest itself. Adapted from Ref[93].

Figure 4. MRP corrects recruitment bias in presidential election polls to provide credible estimates of candidate support

This practical example uses multi-level regression for post-stratification (MRP) to re-weigh the contribution of subgroup-specific data (e.g. from ethnicities and different US states) to an overall estimate (at the level of the whole US) to arrive at a more authentic outcome prediction. This framework addresses the challenge that averages drawn across non-representative participant samples can yield misleading overall outcome predictions, by computationally adjusting for deviations between sample and target population. To estimate political election voting outcomes, views of the Electorate Research Survey interviewed thousands of Americans on 25 June 2020 ahead of the 2020 presidential elections. In total, 6042 persons across 51 states were questioned about their candidate preference. Notably, due to the low sample size, key sociodemographic factors (e.g., ethnicity, sex, education) were highly sampled in a skewed fashion in several states, that is, biased in not faithfully reflecting a state's known population strata with specific combinations of demographic features (demographic slices or 'subgroups' or 'post-stratification cells' in MRP framework). As an example, there were no female respondents in Wyoming. This non-representativeness leads to highly skewed estimates of disaggregation opinions. Disaggregation opinion represents voters' preference percentages disaggregated by state. The estimated support for Biden in Vermont was 73%, while in North Dakota, it was 17%. To counterbalance the imbalanced-stratum-sampling bias, MRP leverages the combined knowledge from external census data (indicating the ground-truth information of subgroup with their combined demographic features) and multilevel regression modeling individual survey responses as a function of demographic and geographic predictors, partially pooling respondents across states to an extent determined by the data. Specifically, the multilevel model estimates for each demographic-geographic respondent type are weighted (post-stratified) by the percentages of each type in the actual state populations. Thus, more representative estimates are obtained for each state (Vermont: 50%, North Dakota 41%). Importantly, the MRP tool can be analogously applied in neuroscience population datasets, such the ABCD cohorts drawn from 21 US states, to achieve representative brain-behavior predictions from a non-representative participant sample at hand. Example reproduced from Ref[94].

Figure 5. High-dimensional visualization tools to explore the hidden subgroups of data points
The neurosciences are generating always more high-dimensional data with input variables in the thousands, such as single cell genomics (snRNAseq) data in this practical example, posing an increasingly urgent need for modern visualization via machine learning tools to discover salient subgroups of data points (here sets of cell transcriptomes with similar expression patterns across typically ~20,000 protein-coding genes), more directly understandable by human intuition. a) Schematic figure demonstrating how stem cells transition into different kinds of cell types and their parallel high-dimensional single-cell transcriptomes, as count matrix (middle), and via machine learning-assisted visualization (PHATE) that recovers the ground-truth cell population subgroups. b) Left, a 2D drawing of an artificial tree with color-coded branches as a ground truth of subgroups (different colors) that should be detected by a pertinent visualization tool. Right, comparison of several such tools (PCA, t-SNE, and PHATE) in how well they recover the known subgroups (colors) in these high-dimensional empirically generated tree data. In this example, PHATE did the best job at detecting the global and local branching structure in this synthetic high-dimensional dataset. c) Comparison of PCA, t-SNE, and the PHATE visualizations for a real-world high-dimensional dataset: embryoid bodies data, with similar trends as in b. d) Here PHATE is deployed in further real data types. Left, human microbiome data shows clear differences between skin, oral, and fecal samples of cell transcriptomes, as well as different enterotypes. Middle, PHATE on Hi-C chromatin conformation data shows the global structure of chromatin. The embedding is colored by the different chromosomes. Right, PHATE on induced stem cell data (iPSC). The embedding is colored by time after induction. Based on the respective merits of these high-dimensional visualization machine learning tools, other forms of complex and many-variable datasets in neuroscience can be explored for biologically meaningful distances between "pockets" of data points in this way. Adapted from Ref[84].

References

1. Sarullo, K. *et al.* Disentangling Socioeconomic Status and Race in Infant Brain, Birth Weight, and Gestational Age at Birth: A Neural Network Analysis. *Biol Psychiatry Glob Open Sci* **4**, 135–144 (2024).
2. Dumornay, N. M., Lebois, L. A. M., Ressler, K. J. & Harnett, N. G. Racial Disparities in Adversity During Childhood and the False Appearance of Race-Related Differences in Brain Structure. *Am J Psychiatry* **180**, 127–138 (2023).
3. Uffelmann, E. *et al.* Genome-wide association studies. *Nat Rev Methods Primers* **1**, 1–21 (2021).
4. Price, A. L. *et al.* Principal components analysis corrects for stratification in genome-wide association studies. *Nat Genet* **38**, 904–909 (2006).
5. Patterson, N., Price, A. L. & Reich, D. Population Structure and Eigenanalysis. *PLOS Genetics* **2**, e190 (2006).
6. Bzdok, D. & Yeo, B. T. T. Inference in the age of big data: Future perspectives on neuroscience. *NeuroImage* **155**, 549–564 (2017).
7. Smith, S. M. & Nichols, T. E. Statistical Challenges in ‘Big Data’ Human Neuroimaging. *Neuron* **97**, 263–268 (2018).
8. Kopal, J., Uddin, L. Q. & Bzdok, D. The end game: respecting major sources of population diversity. *Nat Methods* **20**, 1122–1128 (2023).
9. DeCasien, A. R., Guma, E., Liu, S. & Raznahan, A. Sex differences in the human brain: a roadmap for more careful analysis and interpretation of a biological reality. *Biology of Sex Differences* **13**, 43 (2022).
10. Feczko, E. *et al.* Adolescent Brain Cognitive Development (ABCD) Community MRI Collection and Utilities. 2021.07.09.451638 Preprint at https://doi.org/10.1101/2021.07.09.451638 (2021).

11. Marek, S. *et al.* Reproducible brain-wide association studies require thousands of individuals. *Nature* **603**, 654–660 (2022).
12. Hackman, D. A. & Farah, M. J. Socioeconomic status and the developing brain. *Trends Cogn Sci* **13**, 65–73 (2009).
13. Hackman, D. A., Farah, M. J. & Meaney, M. J. Socioeconomic status and the brain: mechanistic insights from human and animal research. *Nat Rev Neurosci* **11**, 651–659 (2010).
14. Farah, M. J. The Neuroscience of Socioeconomic Status: Correlates, Causes, and Consequences. *Neuron* **96**, 56–71 (2017).
15. Adler, N. E. *et al.* Socioeconomic status and health: The challenge of the gradient. *American Psychologist* **49**, 15–24 (1994).
16. Krieger, N., Williams, D. R. & Moss, N. E. Measuring social class in US public health research: concepts, methodologies, and guidelines. *Annu Rev Public Health* **18**, 341–378 (1997).
17. Yip, S. W., Jordan, A., Kohler, R. J., Holmes, A. & Bzdok, D. Multivariate, Transgenerational Associations of the COVID-19 Pandemic Across Minoritized and Marginalized Communities. *JAMA Psychiatry* **79**, 350–358 (2022).
18. Weaver-Hightower, M. B. Why Education Researchers Should Take School Food Seriously. *Educational Researcher* **40**, 15–21 (2011).
19. Williams, R. Jamie Oliver's school dinners shown to have improved academic results. *The Guardian* (2010).
20. Haworth, S. *et al.* Apparent latent structure within the UK Biobank sample has implications for epidemiological analysis. *Nat Commun* **10**, 333 (2019).
21. Sohail, M. *et al.* Polygenic adaptation on height is overestimated due to uncorrected stratification in genome-wide association studies. *eLife* **8**, e39702 (2019).

22. Solmi, M. *et al.* Age at onset of mental disorders worldwide: large-scale meta-analysis of 192 epidemiological studies. *Mol Psychiatry* **27**, 281–295 (2022).
23. Alvarez, K. *et al.* Race/ethnicity, nativity, and lifetime risk of mental disorders in US adults. *Soc Psychiatry Psychiatr Epidemiol* **54**, 553–565 (2019).
24. Kim, Y. *et al.* Brain-wide Maps Reveal Stereotyped Cell-Type-Based Cortical Architecture and Subcortical Sexual Dimorphism. *Cell* **171**, 456-469.e22 (2017).
25. Bzdok, D. & Meyer-Lindenberg, A. Machine Learning for Precision Psychiatry: Opportunities and Challenges. *Biol Psychiatry Cogn Neurosci Neuroimaging* **3**, 223–230 (2018).
26. Davis, J. *et al.* A review of vulnerability and risks for schizophrenia: Beyond the two hit hypothesis. *Neuroscience and biobehavioral reviews* **65**, 185 (2016).
27. Pearl, J. *Causality: Models, Reasoning, and Inference*. xvi, 384 (Cambridge University Press, New York, NY, US, 2000).
28. Bzdok, D., Nichols, T. E. & Smith, S. M. Towards Algorithmic Analytics for Large-scale Datasets. *Nat Mach Intell* **1**, 296–306 (2019).
29. Bzdok, D., Varoquaux, G. & Steyerberg, E. W. Prediction, Not Association, Paves the Road to Precision Medicine. *JAMA Psychiatry* **78**, 127–128 (2021).
30. Gelman, A., Hill, J. & Vehtari, A. Regression and Other Stories. *Higher Education from Cambridge University Press* https://www.cambridge.org/highereducation/books/regression-and-other-stories/DD20DD6C9057118581076E54E40C372C (2020) doi:10.1017/9781139161879.
31. Wang, Y. & Blei, D. M. The Blessings of Multiple Causes. Preprint at https://doi.org/10.48550/arXiv.1805.06826 (2019).
32. Stoll, C. R. T. *et al.* Multimorbidity in Randomized Controlled Trials of Behavioral Interventions: A Systematic Review. *Health Psychol* **38**, 831–839 (2019).

33. Van Spall, H. G. C., Toren, A., Kiss, A. & Fowler, R. A. Eligibility criteria of randomized controlled trials published in high-impact general medical journals: a systematic sampling review. *JAMA* **297**, 1233–1240 (2007).
34. El Hadi, H., Di Vincenzo, A., Vettor, R. & Rossato, M. Relationship between Heart Disease and Liver Disease: A Two-Way Street. *Cells* **9**, 567 (2020).
35. Tan, Y. Y. *et al.* Comparing clinical trial population representativeness to real-world populations: an external validity analysis encompassing 43 895 trials and 5 685 738 individuals across 989 unique drugs and 286 conditions in England. *The Lancet Healthy Longevity* **3**, e674–e689 (2022).
36. Kopal, J. & Bzdok, D. Endorsing Complexity Through Diversity: Computational Psychiatry Meets Big Data Analytics. *Biol Psychiatry* S0006-3223(22)01479–2 (2022) doi:10.1016/j.biopsych.2022.07.023.
37. Benkarim, O. *et al.* Population heterogeneity in clinical cohorts affects the predictive accuracy of brain imaging. *PLOS Biology* **20**, e3001627 (2022).
38. Westfall, J. & Yarkoni, T. Statistically Controlling for Confounding Constructs Is Harder than You Think. *PLOS ONE* **11**, e0152719 (2016).
39. Di Biase, M. A. *et al.* Mapping human brain charts cross-sectionally and longitudinally. *Proceedings of the National Academy of Sciences* **120**, e2216798120 (2023).
40. Smith, S. M., Vidaurre, D., Alfaro-Almagro, F., Nichols, T. E. & Miller, K. L. Estimation of brain age delta from brain imaging. *NeuroImage* **200**, 528–539 (2019).
41. Zhang, Y., Parmigiani, G. & Johnson, W. E. ComBat-seq: batch effect adjustment for RNA-seq count data. *NAR Genom Bioinform* **2**, lqaa078 (2020).
42. Hastie, T., Tibshirani, R., Friedman, J. H. & Friedman, J. H. *The Elements of Statistical Learning: Data Mining, Inference, and Prediction*. vol. 2 (Springer, 2009).
43. Burton, P. R. *et al.* Genome-wide association study of 14,000 cases of seven common diseases and 3,000 shared controls. *Nature* **447**, 661–678 (2007).

44. Yengo, L. *et al.* A saturated map of common genetic variants associated with human height. *Nature* **610**, 704–712 (2022).

45. Vilhjálmsson, B. J. & Nordborg, M. The nature of confounding in genome-wide association studies. *Nat Rev Genet* **14**, 1–2 (2013).

46. Lander, E. S. & Schork, N. J. Genetic dissection of complex traits. *Science* **265**, 2037–2048 (1994).

47. Menozzi, P., Piazza, A. & Cavalli-Sforza, L. Synthetic maps of human gene frequencies in Europeans. *Science* **201**, 786–792 (1978).

48. Price, A. L., Zaitlen, N. A., Reich, D. & Patterson, N. New approaches to population stratification in genome-wide association studies. *Nat Rev Genet* **11**, 459–463 (2010).

49. Atkinson, E. G. *et al.* Tractor uses local ancestry to enable the inclusion of admixed individuals in GWAS and to boost power. *Nat Genet* **53**, 195–204 (2021).

50. Berg, J. J. *et al.* Reduced signal for polygenic adaptation of height in UK Biobank. *eLife* **8**, e39725 (2019).

51. Boyle, E. A., Li, Y. I. & Pritchard, J. K. An expanded view of complex traits: from polygenic to omnigenic. *Cell* **169**, 1177–1186 (2017).

52. Zeng, J. *et al.* Signatures of negative selection in the genetic architecture of human complex traits. *Nat Genet* **50**, 746–753 (2018).

53. Turchin, M. C. *et al.* Evidence of widespread selection on standing variation in Europe at height-associated SNPs. *Nat Genet* **44**, 1015–1019 (2012).

54. Robinson, M. R. *et al.* Population genetic differentiation of height and body mass index across Europe. *Nat Genet* **47**, 1357–1362 (2015).

55. Barton, N., Hermisson, J. & Nordborg, M. Why structure matters. *eLife* **8**, e45380 (2019).

56. Martin, A. R. *et al.* Clinical use of current polygenic risk scores may exacerbate health disparities. *Nat Genet* **51**, 584–591 (2019).

57. Bureau, U. C. 2020 Census Illuminates Racial and Ethnic Composition of the Country. *Census.gov* https://www.census.gov/library/stories/2021/08/improved-race-ethnicity-measures-reveal-united-states-population-much-more-multiracial.html.
58. Rosenbaum, P. R. & Rubin, D. B. The Central Role of the Propensity Score in Observational Studies for Causal Effects. *Biometrika* **70**, 41–55 (1983).
59. Lee, B. K., Lessler, J. & Stuart, E. A. Improving propensity score weighting using machine learning. *Stat Med* **29**, 337–346 (2010).
60. McCaffrey, D. F., Ridgeway, G. & Morral, A. R. Propensity score estimation with boosted regression for evaluating causal effects in observational studies. *Psychol Methods* **9**, 403–425 (2004).
61. Setoguchi, S., Schneeweiss, S., Brookhart, M. A., Glynn, R. J. & Cook, E. F. Evaluating uses of data mining techniques in propensity score estimation: a simulation study. *Pharmacoepidemiol Drug Saf* **17**, 546–555 (2008).
62. Austin, P. C. An Introduction to Propensity Score Methods for Reducing the Effects of Confounding in Observational Studies. *Multivariate Behav Res* **46**, 399–424 (2011).
63. Rosenbaum, P. R. & Rubin, D. B. Constructing a Control Group Using Multivariate Matched Sampling Methods That Incorporate the Propensity Score. *The American Statistician* **39**, 33–38 (1985).
64. Hernán, M. A., Brumback, B. & Robins, J. M. Marginal structural models to estimate the causal effect of zidovudine on the survival of HIV-positive men. *Epidemiology* **11**, 561–570 (2000).
65. Robins, J. M., Hernán, M. A. & Brumback, B. Marginal structural models and causal inference in epidemiology. *Epidemiology* **11**, 550–560 (2000).
66. Cole, S. R. & Hernán, M. A. Constructing inverse probability weights for marginal structural models. *Am J Epidemiol* **168**, 656–664 (2008).

67. Ali, M. S., Groenwold, R. H. & Klungel, O. H. Best (but oft-forgotten) practices: propensity score methods in clinical nutrition research. *Am J Clin Nutr* **104**, 247–258 (2016).
68. Ali, M. S. *et al.* Propensity Score Methods in Health Technology Assessment: Principles, Extended Applications, and Recent Advances. *Front Pharmacol* **10**, 973 (2019).
69. Cepeda, M. S., Boston, R., Farrar, J. T. & Strom, B. L. Comparison of logistic regression versus propensity score when the number of events is low and there are multiple confounders. *Am J Epidemiol* **158**, 280–287 (2003).
70. Pearl, J. Invited Commentary: Understanding Bias Amplification. *Am J Epidemiol* **174**, 1223–1227 (2011).
71. Gelman, A. & Hill, J. Data Analysis Using Regression and Multilevel/Hierarchical Models. *Higher Education from Cambridge University Press* (2006) doi:10.1017/CBO9780511790942.
72. Gelman, A. *et al. Bayesian Data Analysis*. (Chapman and Hall/CRC, New York, 2015). doi:10.1201/b16018.
73. Gelman, A. & Pardoe, I. Bayesian measures of explained variance and pooling in multilevel (hierarchical) models. *Econometrics* (2004).
74. Kruschke, J. *Doing Bayesian Data Analysis: A Tutorial with R, JAGS, and Stan*. (Academic Press, Boston, 2014).
75. Murphy, K. P. *Machine Learning: A Probabilistic Perspective*. (The MIT Press, Cambridge, MA, 2012).
76. McElreath, R. *Statistical Rethinking: A Bayesian Course with Examples in R and Stan*. (Chapman and Hall/CRC, Boca Raton, 2015).
77. Towards A Principled Bayesian Workflow. https://betanalpha.github.io/assets/case_studies/principled_bayesian_workflow.html#References.

78. Gelman, A. Multilevel (Hierarchical) Modeling: What It Can and Cannot Do. *Technometrics* **48**, 432–435 (2006).
79. Henrich, J., Heine, S. J. & Norenzayan, A. Most people are not WEIRD. *Nature* **466**, 29–29 (2010).
80. Gelman, A. Poststratification into Many Categories Using Hierarchical Logistic Regression. *Survey Methodology* **23**, 127 (1997).
81. Park, D. K., Gelman, A. & Bafumi, J. Bayesian Multilevel Estimation with Poststratification: State-Level Estimates from National Polls. *Political Analysis* **12**, 375–385 (2017).
82. Gao, Y., Kennedy, L., Simpson, D. & Gelman, A. Improving multilevel regression and poststratification with structured priors. *Bayesian Anal* **16**, 719–744 (2021).
83. Wang, W., Rothschild, D., Goel, S. & Gelman, A. Forecasting elections with non-representative polls. *International Journal of Forecasting* **31**, 980–991 (2015).
84. Moon, K. R. *et al.* Visualizing structure and transitions in high-dimensional biological data. *Nat Biotechnol* **37**, 1482–1492 (2019).
85. van Dijk, D. *et al.* Recovering Gene Interactions from Single-Cell Data Using Data Diffusion. *Cell* **174**, 716-729.e27 (2018).
86. Lindenbaum, O., Stanley, J., Wolf, G. & Krishnaswamy, S. Geometry Based Data Generation. in *Advances in Neural Information Processing Systems* vol. 31 (Curran Associates, Inc., 2018).
87. Burkhardt, D. B. *et al.* Quantifying the effect of experimental perturbations at single-cell resolution. *Nat Biotechnol* **39**, 619–629 (2021).
88. Huguet, G. *et al.* A Heat Diffusion Perspective on Geodesic Preserving Dimensionality Reduction. Preprint at https://doi.org/10.48550/arXiv.2305.19043 (2023).
89. Kuchroo, M. *et al.* Single-cell analysis reveals inflammatory interactions driving macular degeneration. *Nat Commun* **14**, 2589 (2023).
90. Novembre, J. *et al.* Genes mirror geography within Europe. *Nature* **456**, 98–101 (2008).

91. Woo, C.-W., Chang, L. J., Lindquist, M. A. & Wager, T. D. Building better biomarkers: brain models in translational neuroimaging. *Nat Neurosci* **20**, 365–377 (2017).
92. Schoeler, T. *et al.* Participation bias in the UK Biobank distorts genetic associations and downstream analyses. *Nat Hum Behav* **7**, 1216–1227 (2023).
93. Bonkhoff, A. K. *et al.* Bringing proportional recovery into proportion: Bayesian modelling of post-stroke motor impairment. *Brain* **143**, 2189–2206 (2020).
94. Li, S. Forecasting the 2020 US Election Using Multilevel Regression with Post-stratification. *Medium* https://towardsdatascience.com/forecasting-the-2020-us-election-using-multilevel-regression-with-post-stratification-bbf2f93585fb (2021).

Figure

Click here to access/download;Figure;fig1.pdf

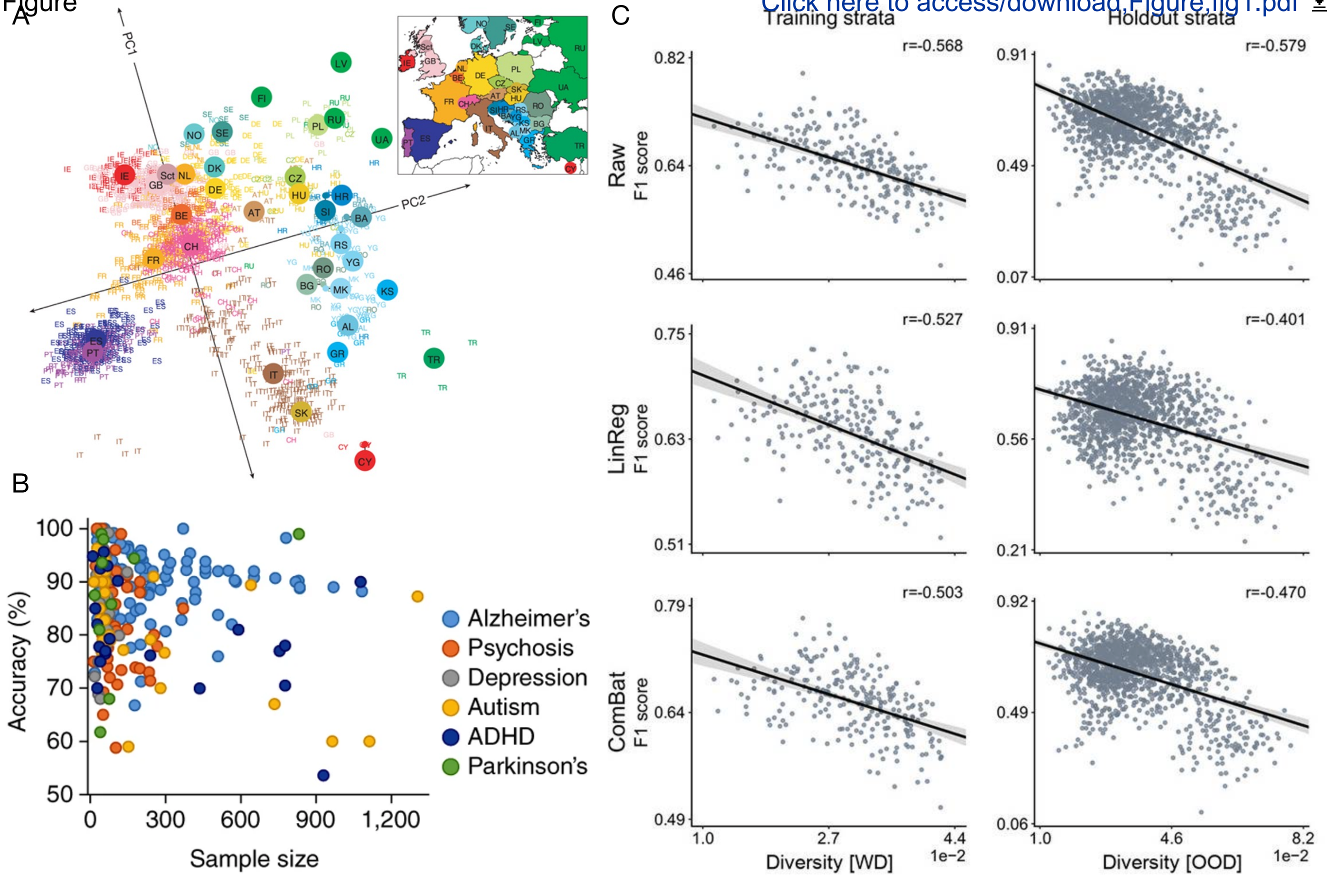

Figure

Click here to access/download;Figure;fig2.pdf

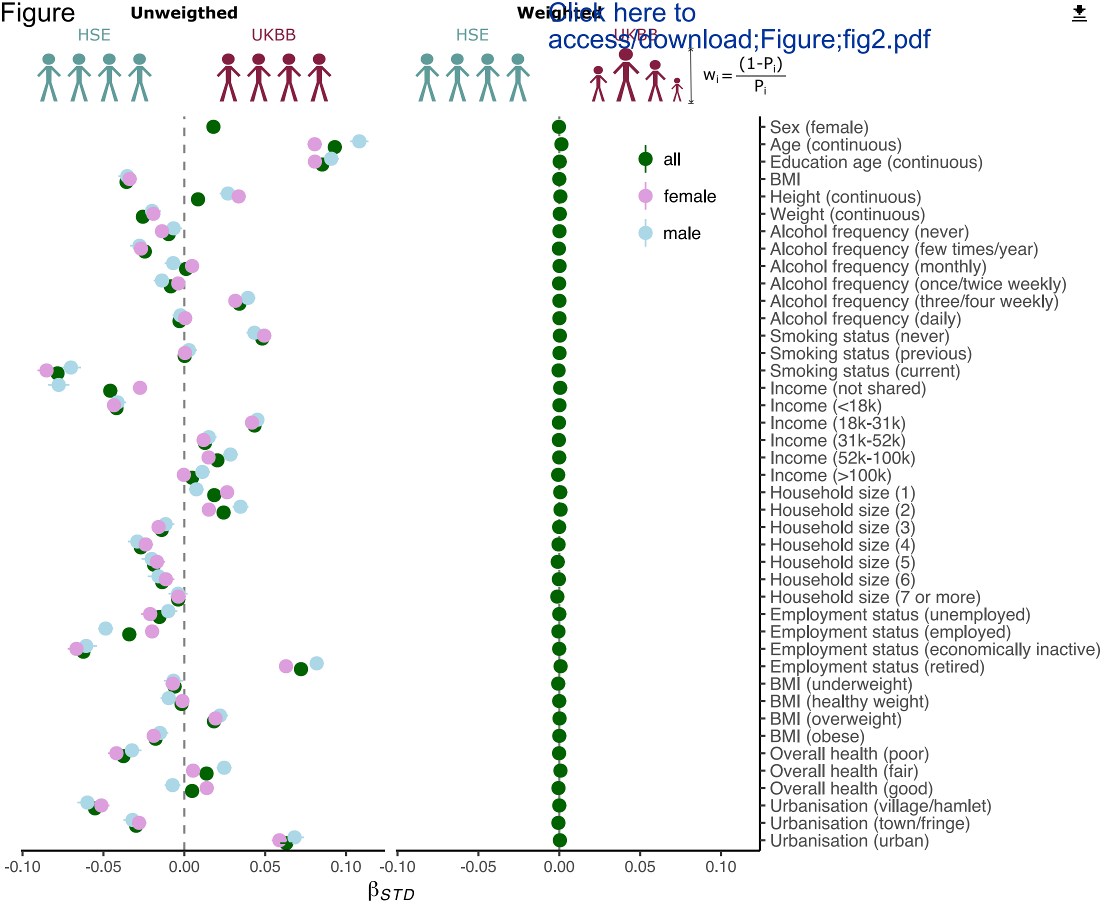

Figure
Click here to access/download;Figure;fig3.pdf

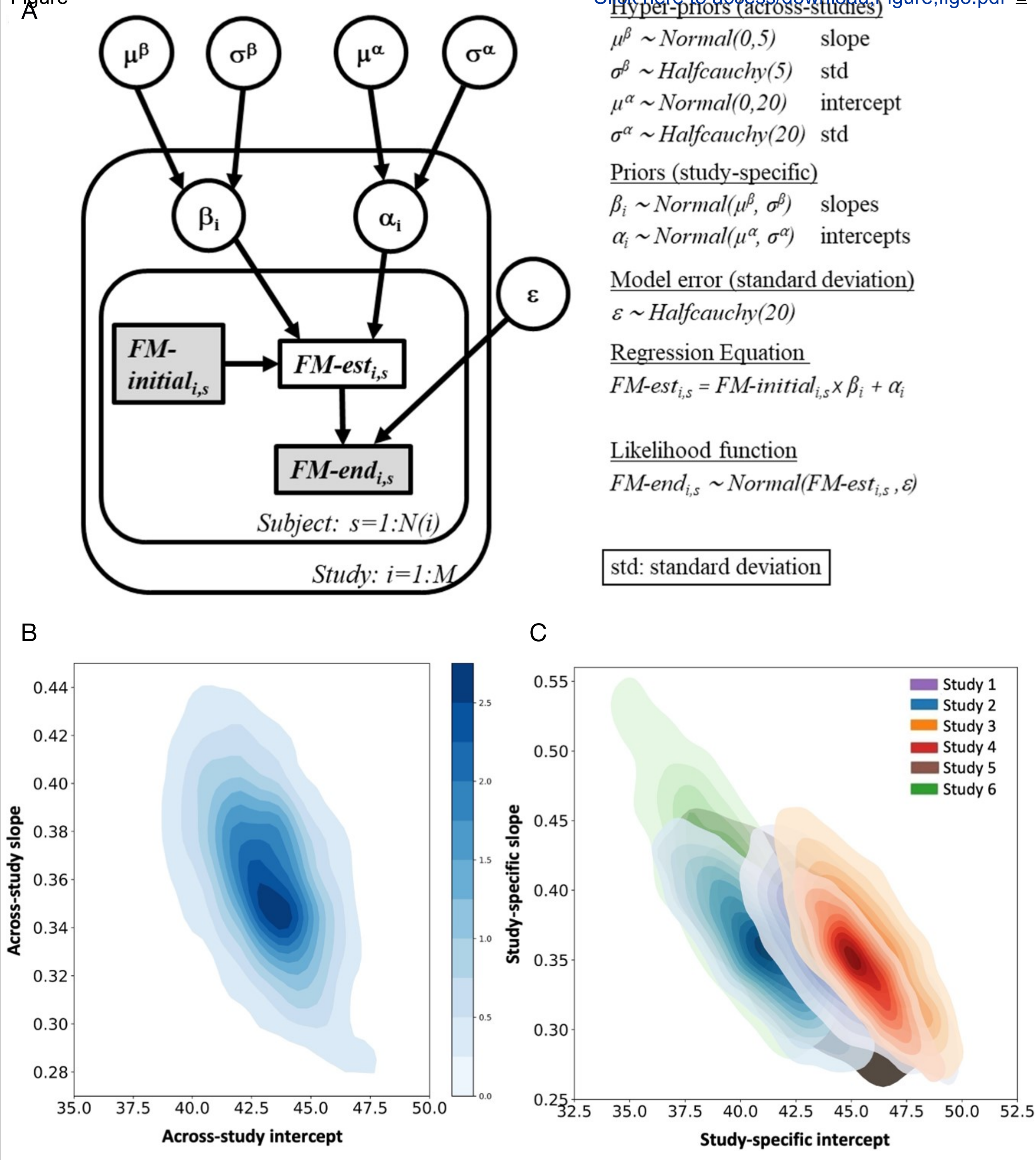

Figure

Click here to access/download;Figure;fig4.pdf

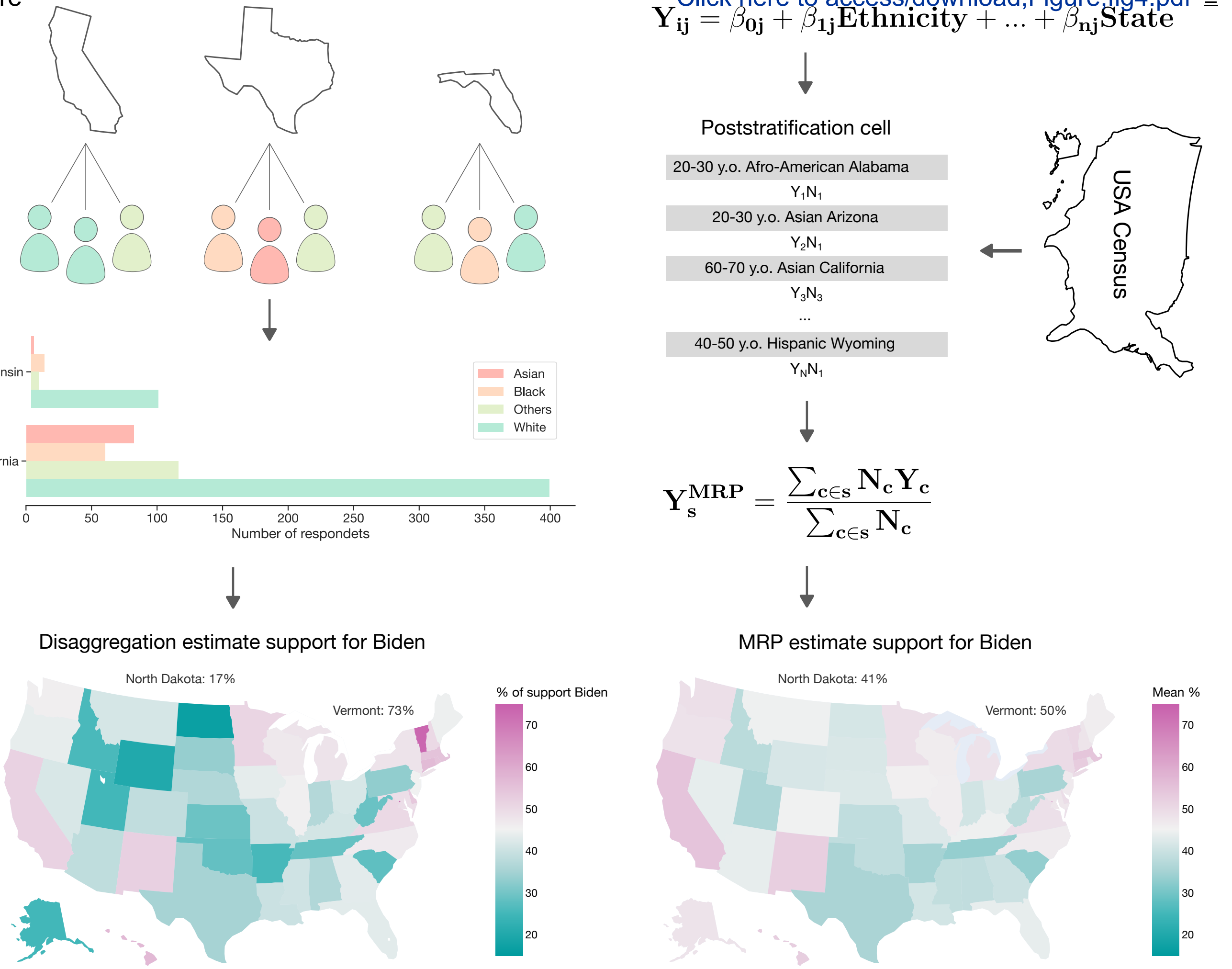

Figure
Click here to access/download;Figure;fig5.pdf

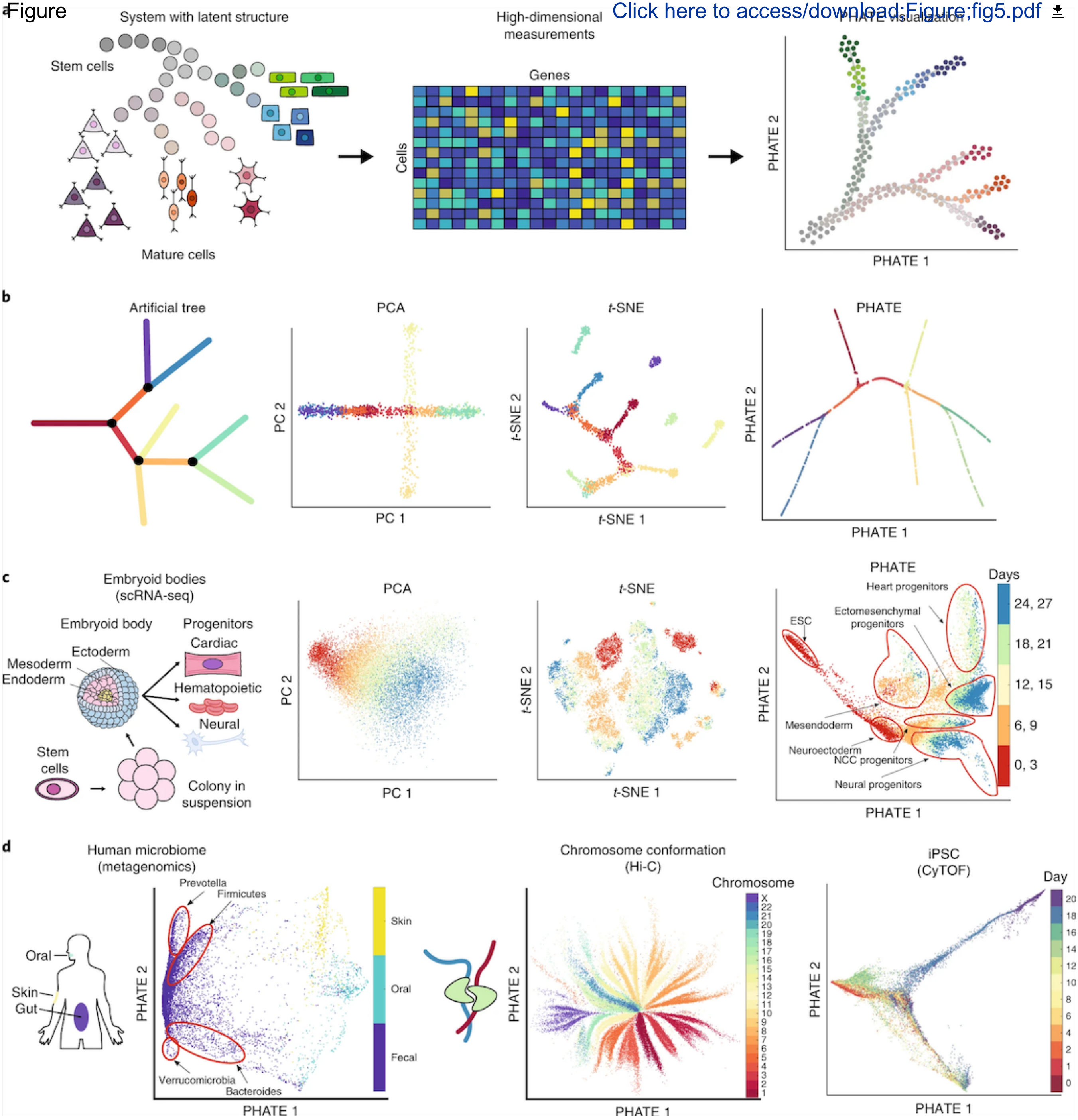

Cover letter

Click here to access/download;Personal Cover;cover_240609.docx

Institut et hôpital neurologiques de Montréal
Montreal Neurological Institute and Hospital

Danilo Bzdok, MD, PhD
Montreal Neurological Institute
3801 rue University, Bureau #872D
Montréal (Québec) H3A 2B4
Canada

GigaScience, Members of the Editorial Board

Harnessing population diversity: In search for tools of the trade

Danilo Bzdok[1,2], Guy Wolf[1], Jakub Kopal[1,2]

[1]*MNI – Montreal Neurological Institute, Montreal*
[2]*MILA – Quebec Artificial Intelligence Institute, Montreal*

Historically, the neurosciences have been the study of WEIRD brains. Despite making up only 12% of the world's population, Western, Educated, Industrial, Rich, and Democratic (WEIRD) participants form a majority of studied neuroscientific cohorts. This apparent discrepancy did not arise because neuroscientists globally worked to omit 88 % of the world's population, but it's instead the product of recruiting subject samples of convenience. In other words, human neuroscience studies, including brain-scanning and GWAS studies, are very expensive, so most of them are small and rely on a thin and unrepresentative slice of humanity. In many areas of neuroscience, boutique datasets acquired in-house remain central for the modus operandi. Concretely, almost 90% of recent studies used self-acquired datasets. Moreover, more than half of these datasets included sample sizes of <24 participants. Critically, these clinical data collection initiatives often come with narrow and shallow phenotyping – typically restricted to participant age and biological sex. Such modest phenotyping stands in sharp contrast to emerging large population-based cohorts of thousands of participants and their deep phenotypic profiling. Hence, population datasets are poised to improve our ability to predict outcomes in individual participants. However, recent work highlights how algorithmic biases favor majority populations, and predictive models often fail to generalize to minority populations. The success of our modeling tools for single-subject predictions is interlocked with population diversity, including demographic differences and other factors that may be currently under-appreciated.

Key arguments why neuroscience is at a crossroads:

- Major sources of population stratification are often stronger than effects of interest; subtle brain and cognitive effects get overwhelmed by sources of variation that are not of primary scientific interest; in brain-imaging, height, weight, and BMI routinely show much stronger effects in the brain measures than what neuroscience studies set out to interrogate; in genetics, genetic ancestry is a stronger force than the typically low-effect single nucleotide polymorphisms that GWAS uncovers even in traits such as intelligence and educational attainment
- Autism spectrum disorder, schizophrenia, or Alzheimer's disease exhibit subpopulation-related differences in prevalence, symptoms, and treatment response. Major brain disorders need to be investigated in a diverse pool of participants since these disorders have complex mechanisms that are, in many cases, interlocked with sex, age, ethnicity, and potentially many social identity factors
- Consequence: predictive models formed on brain data dominated by white participants break down in their performances when used to predict phenotypes for Black participants in the Human Connectome Project and ABCD

- => Identifying sociodemographic factors intertwined with the target brain-behavior relationship opens the door to refined and more accurate phenotype predictions
- => Sources of population diversity are bigger driving forces in the various kinds of brain-derived data than the subtle effects of interest that neuroscientists are usually after

Why today's practices for confound variable correction are insufficient:
- Historically, our deconfounding practices mostly come from a time when our participant samples were much more homogenous samples from a local geography (e.g., investigator's university students) and/or with strict inclusion/exclusion criteria that – in both scenarios – narrowed down the spread of the target sample under investigation
- HCP, UK Biobank, ENIGMA and other endeavors set out only a few years ago and provide, perhaps for the first time in neuroscience, nuanced indicators tracking each participants diversity indicators and sociodemographic position in society
- We can only account for nuisance sources that we have measured; but known unknown like differences in various demographic dimensions are not available in many datasets, including several of the most widely used ones (cf. ignorability assumption)
- We are only correcting for linear variation, but not non-linear structure; hence, any more advanced downstream modeling tools, such as many machine learning algorithms and deep learning will still pick up the nuisance variable, but their more complex patterns
- More broadly, deconfounding modeling practices are often idiosyncratic to a particular lab or research pocket or neuroscience field ("we do it this way because that is how the others are doing it"); e.g. in the RNAseq community people use other deconfounding techniques every day than imaging neuroscience (typically two step processing, first deconfounding, then running statistical model of interest) or GWAS genetics (typically, confound variables are in the model of interest)
- **=> Importantly, a large dataset may not be a big small dataset in terms of appropriate modeling practices**

Candidate tool: Propensity scores
- Trusted in certain research communities, such as epidemiology, but rarely used in neuroscience
- Once we have more than two or three covariates of no interest (e.g. age, sex and IQ), manual matching of participants, probably the most common matching approach in neuroscience today, is not typically straightforward or outright infeasible, especially when combining yes/no and continuous variables
- Allows for balanced comparisons between partially non-overlapping groups of participants
- We want to restructure our comparison group to *look like* our treatment group regarding the distribution of observed covariates; create groups that have similar distributions of confounding variables
- Advantages: allows for one-number summary of a set of heterogeneous covariates; a non-linear propensity score model can be used in principle to catch more complex confounding structure in the data; explicit frameworks for measuring and tackling the degree of overlap between groups
- Disadvantages: originally framed in treatment-control setting, but more widely applicable
- Example from neuroscience: PS were recently used to show that site factors drive results in autism classification models in ABIDE (Benkarim et al., 2021)
- => Can achieve refined population matching based on many diversity factors

Candidate tool: Bayesian hierarchical modeling
- One of the best statistical tools to seamlessly integrate heterogeneous sources of information in an integrated framework; although neuroscience has used Bayesian techniques for modeling and

as inspiration for cognitive models, applications for advanced confound modeling remains surprisingly under-explored

- Perfectly adapted to highly structured datasets of which we have more and more in different neuroscience areas (in contrast to in-lab experiments of locally recruited subjects, with only age and sex as meta-information)
- Can continuously estimate how much sharing of statistical information between groups is appropriate; also by integrating existing domain knowledge (base rates in the population or fractions between race composition)
- Classical linear regression (deconfounding) models ascribe no structure to the parameters; the future my lie in adding population structure to priors in dedicated regression models
- Advantages: Explicit generative modeling of various different confounding variables and their mutual relationships; Principled quantification of uncertainty (estimating the clean contours of uncertainty of a kind rarely available in non-Bayesian approaches); Borrowing statistical strength from small or under-represented groups in a principled modeling mechanism; capable of stating and appropriately handling massive confound interactions and multiple levels of them; accounting for individual and group-level variation in estimating group-level coefficients
- Disadvantages: Need to be hand-tailored to each particular modeling setting, which requires technical and mathematical expertise in the project team; computationally more expensive (but cheaper than estimating many deep learning tools)
- => Building hierarchical population models to pool information across groups in the data to produce better estimates for all groups

Candidate tool: Multilevel regression and post-stratification ("MR-P")

- Used in political polling and election prediction (first publication 1997), but we are not aware of usage in the neuroscience community (our lab has ongoing projects for inter-community translation)
- A statistical technique used for estimating preferences in sub-regions (e.g., states, individual constituencies) based on individual-level survey data gathered at other levels of aggregation (e.g., national surveys)
- Can systematically adjust for discrepancies between sample and target population: Makes sub-models for distinct population strata first, and then can rebalance the stratum-specific predictions to "un-skew" by correcting for the particular sampling demographics
- Advantages: Explicitly quantify and alleviate the bias in predictions from neuroscience data; Can integrate external knowledge such as population characteristics available through CENSUS in US and Canada
- Disadvantages: Cannot account for all forms of selection bias, but many common ones of the type that we see in UK Biobank, ABCD and other emerging population cohorts
- **=> Systematic modeling tool to de-bias single-subject predictions in under-represented and under-sampled groups**

Candidate tool: Machine-learning algorithms

- Misaligned interests: as a whole, the machine learning community is less interested in careful variable selection, significant single-variable effects and confound removal
- Standard linear regression deconfounding can be replaced by more advanced non-linear regression methods from machine learning; explain what these data modeling settings are
- However, there are recent algorithmic innovations that can be used for the purpose of explicitly modeling of population diversity

- As a general trend, ML algorithms may turn out to be more useful for charting major sources of population stratification and visualizing it (e.g., using tools such as t-SNE, UMAP); rather than, directly accounting for confounding influences in analyses of primary scientific interest
- Example: PHATE as a diffusion-model based approach to improve and nuance our visualization approaches to detect subtle trends in high-dimensional data such as single-cell gene expressions
- Example: condition Variational auto-encoders were used in a few seminal studies in RNAseq transcriptomics
- => Perhaps machine learning algorithms will turn out to mostly help delineating and visualizing the still under-explored major sources of population stratification, rather than helping to explicit address and alleviate there influence on quantities of primary scientific interest, once discovered

References

- Benkarim, O., Paquola, C., Park, B., Kebets, V., Hong, S.-J., Wael, R.V. de, Zhang, S., Yeo, B.T.T., Eickenberg, M., Ge, T., Poline, J.-B., Bernhardt, B.C., Bzdok, D., 2022. Population heterogeneity in clinical cohorts affects the predictive accuracy of brain imaging. PLOS Biol. 20, e3001627.
- Bonkhoff AK, …, Bzdok D, Wu O, Rost NS. Sex-specific lesion pattern of functional outcomes after stroke, Brain Communications, 2022.
- Bzdok, D., Krzywinski, M., Altman, N., 2018. Machine learning: supervised methods. Nat. Methods 15, 5–6.
- Bzdok, D., Meyer-Lindenberg, A., 2018. Machine Learning for Precision Psychiatry: Opportunities and Challenges. Biol Psychiatry Cogn Neurosci Neuroimaging 3, 223–230.
- Clemens B, Derntl B, Smith E, Junger J, Neulen J, Mingoia G, Schneider F, Abel T, Bzdok D*, Habel U*. Predictive pattern classification can distinguish gender identity subtypes from behavior and brain imaging. Cerebral Cortex, 2020.
- Dockès, J., Varoquaux, G., Poline, J.-B., 2021. Preventing dataset shift from breaking machine-learning biomarkers. GigaScience 10, giab055.
- Falk, E.B., Hyde, L.W., Mitchell, C., Faul, J., Gonzalez, R., Heitzeg, M.M., Keating, D.P., Langa, K.M., Martz, M.E., Maslowsky, J., Morrison, F.J., Noll, D.C., Patrick, M.E., Pfeffer, F.T., Reuter-Lorenz, P.A., Thomason, M.E., Davis-Kean, P., Monk, C.S., Schulenberg, J., 2013. What is a representative brain? Neuroscience meets population science. Proc. Natl. Acad. Sci. U. S. A. 110, 17615–17622. https://doi.org/10.1073/pnas.1310134110
- Henrich, J., Heine, S. & Norenzayan, A. Most people are not WEIRD. Nature 466, 29 (2010).
- Kandel, E.R., Markram, H., Matthews, P.M., Yuste, R., Koch, C., 2013. Neuroscience thinks big (and collaboratively). Nat. Rev. Neurosci. 14, 659–664.
- Kaplan, R.M., Chambers, D.A., Glasgow, R.E., 2014. Big Data and Large Sample Size: A Cautionary Note on the Potential for Bias. Clin. Transl. Sci. 7, 342–346.
- Kweon H, Aydogan G, Dagher A, Bzdok D, Ruff CC, Nave G, Farah MJ, Koellinger PD. Human brain anatomy reflects separable genetic and environmental components of socioeconomic status, Science Advances, 2022.
- Kiesow H, Uddin LQ, Bernhardt BC, Kable JW, Bzdok D. Dissecting the midlife crisis: Disentangling social, personality and demographic determinants in social brain anatomy, Communications Biology, Nature Publishing Group, 2021.
- Kievit, R. A., Frankenhuis, W. E., Waldorp, L. J., & Borsboom, D. (2013). Simpson's paradox in psychological science: a practical guide. Frontiers in psychology, 4, 513.

- Li, J., Bzdok, D., Chen, J., Tam, A., Ooi, L.Q.R., Holmes, A.J., Ge, T., Patil, K.R., Jabbi, M., Eickhoff, S.B., Yeo, B.T.T., Genon, S., 2022. Cross-ethnicity/race generalization failure of behavioral prediction from resting-state functional connectivity. Sci. Adv. 8, eabj1812.
- Poeppl T, Dimas E, Sakreida K, Kernbach J, Markello R, Schoeffski O, Dagher A, Koellinger P, Nave G, Farah M, Misic B, Bzdok D. Pattern learning reveals brain asymmetry to be linked to socioeconomic status, Cerebral Cortex Communications, 2022.
- Szucs, D., Ioannidis, J.PA., 2020. Sample size evolution in neuroimaging research: An evaluation of highly-cited studies (1990–2012) and of latest practices (2017–2018) in high-impact journals. NeuroImage 221, 117164.
- Uddin, L.Q., De Los Reyes, A., 2022. Developmental Considerations for Understanding Perceptions and Impacts of Identity-Related Differences: Focusing on Adolescence. Biol Psychiatry Cogn Neurosci Neuroimaging 7, 1209–1214.
- Webb, E.K., Etter, J.A. & Kwasa, J.A. Addressing racial and phenotypic bias in human neuroscience methods. Nat Neurosci 25, 410–414 (2022).
- Woo, C.W., Chang, L.J., Lindquist, M.A., Wager, T.D., 2017. Building better biomarkers: brain models in translational neuroimaging. Nat. Neurosci. 20, 365–377.
- Yip S*, Jordan A*, Kohler RJ, Holmes A, Bzdok D. Multivariate, transgenerational associations of the COVID-19 pandemic across minoritized and marginalized communities, JAMA Psychiatry, 2022.
