# Supplementary material for: Harnessing population diversity: in search of tools of the trade
Source: Gigascience. 2024 Sep 27;13:giae068. doi: 10.1093/gigascience/giae068 (PMC11427908; doi:10.1093/gigascience/giae068)
Supplement: giae068_Response_to_Reviewer_Comments_Original_Submission [file giae068_response_to_reviewer_comments_original_submission.pdf]

Author's Response To Reviewer Comments

Reviewer reports:
Reviewer #1: This manuscript addresses an important challenge in neuroscience - the use of computational tools to enhance the inclusion of diverse populations in human neuroscience research. The piece offers valuable insights for researchers in this field. Below, I have outlined several points for the authors to consider.

We are grateful to the reviewer for the positive assessment of our work. We have followed the recommendations of the reviewers that led to an improved version of our manuscript. We here provide point-by-point answers to all raised comments. Deleted parts of the original text are highlighted in red, and added parts in blue below. A complete set of edits is available in the attached manuscript with full track changes.

Major comments:
I commend the authors for integrating connections to genetic approaches throughout the manuscript. However, the paper would benefit from a clearer definition of what constitutes "diverse backgrounds" and how diversity is represented within a sample. The current usage of "diverse backgrounds" is somewhat ambiguous, as diversity refers to populations rather than individuals. Phrasing such as "wider diversity backgrounds" could be more accurately expressed as "wider, diverse backgrounds." A more precise explanation of the authors' interpretation of diversity would enhance the clarity of the manuscript.

We thank the reviewer for highlighting the potential lack of clarity on our side. We unequivocally agree that groups or cohorts are sampled from diverse backgrounds, but a single person is not diverse. As a response to the reviewer's suggestion, we now provide a precise definition of the term “diversity background”. In addition, we inspected the entire manuscript to ensure consistent and accurate usage of the term throughout the text, making the distinction between individual characteristics and group-level diversity more explicit. Here we list the changes:

Abstract:
In our opinion, as we sample individuals from always wider diversity wider, diverse backgrounds, we will require a new stack of quantitative tools to realize diversity-aware modeling.

Line 132:
The notion of ‘diversity’ here follows the empirical observation that age, sex and ethnicity (as commonly available in many biomedical dataset) are an incomplete standard for describing an individual's position in the fabric of society.

Line 155:
Perhaps for the first time in the history of neuroscience, these collections provide sufficiently detailed and nuanced pictures of each participant’s diversity background, including the sociodemographic anchoring in the wider society28 (i.e., the combination of demographic and identity factors28).

Line 661:
As we pull subjects from always more geographies and sociological pockets, and diversity backgrounds, the inconsistencies in what sources of major population stratification are acknowledged and integrated explicitly into our everyday modeling practices become center stage.

The paragraphs on lines 91-99 and 100-105 seem slightly out of place. The discussion shifts from best practices related to COVID-19 to studies on food and nutrition. While I understand the authors' intent, the transition is not entirely seamless. It may be more effective to either combine these paragraphs into one or remove the second paragraph. Alternatively, the authors could emphasize the interdisciplinary nature of these topics, acknowledging the interconnectedness of these issues and the fact that individuals do not exist in a vacuum.

We thank the reviewer for the suggestion. We removed the second paragraph in order to improve the flow of the text.

Line 100:
Therefore, dimensions of social inequality played an incisive role in the consequences of the lockdown experience imposing mass social isolation – which should be taken into account in brain-behavior studies with recruiting during COVID-19.
At the level of nutrition habits, the food environment can color and confound study results18. As a “natural experiment”, several schools in Greenwich, England, participated in a campaign to replace highly processed food high in fat, salt, and sugar with healthier meals prepared from scratch19. Providing healthier food resulted in improvements in literacy and science tests. Therefore, nutrition and other environmental factors can introduce skewing into brain-behavior studies, if unacknowledged, thus deteriorating their conclusions’ validity and generalizability

Given the comments above, and the ongoing uncertainty regarding which dimensions of population diversity are most critical when modeling phenotypes, I recommend incorporating additional examples or literature that address the limitations of deep phenotyping. Specifically, it would be beneficial to discuss how, despite comprehensive phenotyping efforts, certain individuals or groups may still be underrepresented or overlooked in these studies. This could provide a more comprehensive perspective on the issues discussed.

We thank the reviewer for highlighting this very important topic. In response, we have incorporated additional literature that highlights the challenges associated with deep phenotyping, specifically addressing how certain individuals or groups may still be underrepresented or overlooked despite comprehensive phenotyping efforts. For instance, we now discuss studies that emphasize the need for careful consideration of sociodemographic factors, such as race, ethnicity, and socioeconomic status, in deep phenotyping approaches, citing specific examples where certain subgroups in the populations remain underrepresented despite large-scale recruitment efforts (e.g., Dick et al., 2021; Fry et al., 2017). These additions help illustrate the inherent limitations and ongoing challenges in achieving truly representative and diverse cohorts.

Line 175:
That is why population datasets are an untapped asset, or perhaps even a necessary prerequisite, to be in a position to start unraveling the form and implications of major diversity factors for our phenotypes of actual interest29.
It is important to note that despite comprehensive phenotyping efforts, certain individuals or groups may still be underrepresented or overlooked even in large population datasets (Kopal et al., 2023). In the UK Biobank, non-European ethnic groups are significantly underrepresented (Fry et al, 2015). Similarly, and despite efforts, some racial and ethnic groups are still underrepresented in the ABCD dataset. From a broader perspective population datasets tend to have a higher representation of participants from higher socioeconomic backgrounds that are healthier than their population averages, while participants from rural or remote areas as well as participants with physical or cognitive disabilities are often underrepresented. Despite these challenges, the recent advent of big data in neuroscience6 thus puts us in a position where we can start developing new methods and frameworks able to identify dimensions of population diversity and incorporate them appropriately in everyday modeling efforts.

Minor Comments:
- Lines 179-180: The statement, "Historically, there has perhaps been an overreliance on convenience-based recruitment of study participants in neuroscience," requires a citation.

We added the missing citation:
Henrich J, Heine SJ, Norenzayan A. The weirdest people in the world? Behavioral and Brain Sciences. 2010;33(2-3):61-83. doi:10.1017/S0140525X0999152X

Line 186:
Historically, there has perhaps been an overreliance on convenience-based recruitment of study participants in neuroscience (Henrich et al., 2010). We inherited our deconfounding practices mainly from a time when our participant samples were homogenous samples drawn from a local geography, a single city, or students from a single university department or patients admitted to only one medical clinic.

- Line 92: The phrase "experience of ..." should be revised to "experiences of ..."

Corrected as suggested.

Line 93:
At the level of extraordinary population events, sociological factors such as immigration status, mother tongue, single-parenthood, and experiences of racism emerged as the primary contributors to negative experiences during the COVID-19 pandemic.

- abstract: "each individual's positionin" should be "positioning".

We refined the sentence for clarity and flow.

Abstract:
Neuroscience has now witnessed the advent of many population cohort studies that deep-profile participants, yielding hundreds of measures, capturing dimensions of each individual’s position in the broader society.
Neuroscience has now witnessed the advent of many population cohort studies that deeply profile participants, yielding hundreds of measures that capture various dimensions of each individual’s position within broader society.
