# Supplementary material for: Harnessing population diversity: in search of tools of the trade
Source: Gigascience. 2024 Sep 27;13:giae068. doi: 10.1093/gigascience/giae068 (PMC11427908; doi:10.1093/gigascience/giae068)
Supplement: giae068_Reviewer_1_Report_Original_Submission — Jocelyn Ricard -- 8/12/2024 Reviewed [file giae068_reviewer_1_report_original_submission.pdf]

Reviewer Report

Title: Harnessing population diversity: In search for tools of the trade

Version: Original Submission **Date:** 8/12/2024

Reviewer name: Jocelyn Ricard

Reviewer Comments to Author:

This manuscript addresses an important challenge in neuroscience - the use of computational tools to enhance the inclusion of diverse populations in human neuroscience research. The piece offers valuable insights for researchers in this field. Below, I have outlined several points for the authors to consider.
Major comments:
I commend the authors for integrating connections to genetic approaches throughout the manuscript. However, the paper would benefit from a clearer definition of what constitutes "diverse backgrounds" and how diversity is represented within a sample. The current usage of "diverse backgrounds" is somewhat ambiguous, as diversity refers to populations rather than individuals. Phrasing such as "wider diversity backgrounds" could be more accurately expressed as "wider, diverse backgrounds." A more precise explanation of the authors' interpretation of diversity would enhance the clarity of the manuscript.
The paragraphs on lines 91-99 and 100-105 seem slightly out of place. The discussion shifts from best practices related to COVID-19 to studies on food and nutrition. While I understand the authors' intent, the transition is not entirely seamless. It may be more effective to either combine these paragraphs into one or remove the second paragraph. Alternatively, the authors could emphasize the interdisciplinary nature of these topics, acknowledging the interconnectedness of these issues and the fact that individuals do not exist in a vacuum.
Given the comments above, and the ongoing uncertainty regarding which dimensions of population diversity are most critical when modeling phenotypes, I recommend incorporating additional examples or literature that address the limitations of deep phenotyping. Specifically, it would be beneficial to discuss how, despite comprehensive phenotyping efforts, certain individuals or groups may still be underrepresented or overlooked in these studies. This could provide a more comprehensive perspective on the issues discussed.
Minor Comments:
- Lines 179-180: The statement, "Historically, there has perhaps been an overreliance on convenience-based recruitment of study participants in neuroscience," requires a citation.
- Line 92: The phrase "experience of ..." should be revised to "experiences of ..."
- abstract: "each individual's positionin" should be "positioning".

Methods

Are the methods appropriate to the aims of the study, are they well described, and are necessary controls included? Choose an item.

Conclusions

Are the conclusions adequately supported by the data shown? Choose an item.

Reporting Standards

Does the manuscript adhere to the journal's guidelines on minimum standards of reporting? Choose an item.

Choose an item.

Statistics

Are you able to assess all statistics in the manuscript, including the appropriateness of statistical tests used? Choose an item.

Quality of Written English

Please indicate the quality of language in the manuscript: Choose an item.

Declaration of Competing Interests

Please complete a declaration of competing interests, considering the following questions:

- Have you in the past five years received reimbursements, fees, funding, or salary from an organisation that may in any way gain or lose financially from the publication of this manuscript, either now or in the future?
- Do you hold any stocks or shares in an organisation that may in any way gain or lose financially from the publication of this manuscript, either now or in the future?
- Do you hold or are you currently applying for any patents relating to the content of the manuscript?
- Have you received reimbursements, fees, funding, or salary from an organization that holds or has applied for patents relating to the content of the manuscript?
- Do you have any other financial competing interests?
- Do you have any non-financial competing interests in relation to this paper?

If you can answer no to all of the above, write 'I declare that I have no competing interests' below. If your reply is yes to any, please give details below.

No

I agree to the open peer review policy of the journal. I understand that my name will be included on my report to the authors and, if the manuscript is accepted for publication, my named report including any attachments I upload will be posted on the website along with the authors' responses. I agree for my

report to be made available under an Open Access Creative Commons CC-BY license (http://creativecommons.org/licenses/by/4.0/). I understand that any comments which I do not wish to be included in my named report can be included as confidential comments to the editors, which will not be published.

Choose an item.

To further support our reviewers, we have joined with Publons, where you can gain additional credit to further highlight your hard work (see: https://publons.com/journal/530/gigascience). On publication of this paper, your review will be automatically added to Publons, you can then choose whether or not to claim your Publons credit. I understand this statement.

Yes Choose an item.
